# Ophthalmic Sensors and Drug Delivery

**DOI:** 10.1021/acssensors.1c00370

**Published:** 2021-05-27

**Authors:** Antonysamy Dennyson Savariraj, Ahmed Salih, Fahad Alam, Mohamed Elsherif, Bader AlQattan, Ammar A. Khan, Ali K. Yetisen, Haider Butt

**Affiliations:** † Department of Mechanical Engineering, Khalifa University of Science and Technology, Abu Dhabi, United Arab Emirates; ‡ Department of Physics, Lahore University of Management Sciences, Lahore Cantonment 54792, Lahore, Pakistan; § Department of Chemical Engineering, Imperial College London, London SW7 2AZ, United Kingdom

**Keywords:** ophthalmology, contact lenses, continuous monitoring, physiological parameters, biosensors, biomaterials, photonic crystals, bioavailability, diagnostics, personalized medicine, drug delivery

## Abstract

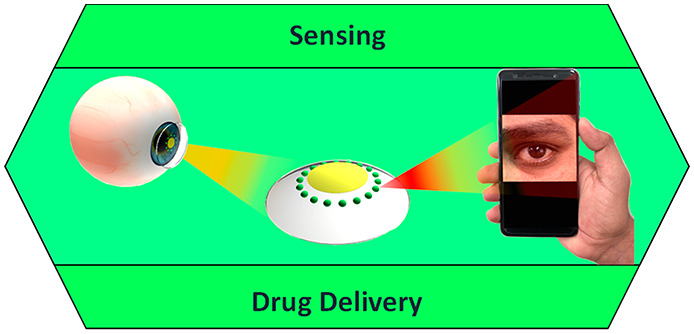

Advances in multifunctional materials and technologies have allowed contact lenses to serve as wearable devices for continuous monitoring of physiological parameters and delivering drugs for ocular diseases. Since the tear fluids comprise a library of biomarkers, direct measurement of different parameters such as concentration of glucose, urea, proteins, nitrite, and chloride ions, intraocular pressure (IOP), corneal temperature, and pH can be carried out non-invasively using contact lens sensors. Microfluidic contact lens sensor based colorimetric sensing and liquid control mechanisms enable the wearers to perform self-examinations at home using smartphones. Furthermore, drug-laden contact lenses have emerged as delivery platforms using a low dosage of drugs with extended residence time and increased ocular bioavailability. This review provides an overview of contact lenses for ocular diagnostics and drug delivery applications. The designs, working principles, and sensing mechanisms of sensors and drug delivery systems are reviewed. The potential applications of contact lenses in point-of-care diagnostics and personalized medicine, along with the significance of integrating multiplexed sensing units together with drug delivery systems, have also been discussed.

Effective health care can only be realized through effective medical detection, diagnosis, and treatment.^1^ To achieve such a goal, internal parameters are to be continuously monitored to ensure prompt diagnosis of diseases and to avail prompt treatments to critically ill patients. Diabetes mellitus and glaucoma are two important health disorders that can cause irreversible vision loss. Moreover, their symptoms remain dormant even in advanced stages and one has to closely monitor them to avoid adverse health complications. Monitoring glucose concentrations and intraocular pressure (IOP) are important in the diagnosis and treatment of diabetes mellitus and glaucoma, respectively. The routinely employed traditional tools and technologies are often unsuccessful, failing to meet the demands in terms of non-invasive continuous monitoring, a vital feature in the point-of-care testing and personalized medicine. Besides that, using invasive and tethered devices and frequent laboratory visits are so inconvenient that periodic testing is interrupted. Regular monitoring of glucose concentration and intraocular pressure can help to determine the long-term fluctuations in patients^2^ and to prescribe adequate medication.

Recent progress in multifunctional materials and technologies have paved the way for the development of portable devices whose diagnostic competencies excel compared to existing technologies. Taking advantage of the self-examination feature and portability, tiny sized sensors find their way in the examination of different samples such as blood, urine, and sweat; nevertheless, the inconveniences related to sample collection and vulnerability to contamination definitely necessitate a better alternative. Tear fluid is an equally rich source of several biomarkers and can be procured uninterruptedly compared to other samples. So tear fluid becomes potentially a better sample to continuously monitor physiological parameters^1^ such as concentration of tear glucose,^3^ urea, proteins, nitrite,^4^ and chloride ions,^5^ IOP,^6^ corneal temperature,^7^ moisture,^8^ and pH.^9^ However, upon realizing the adverse health damage caused by diabetes mellitus and glaucoma, they are to be given the foremost priority in diagnostics. To realize tear fluid examination for the diagnosis of diabetes and glaucoma, contact lenses become a viable platform to carry out both diagnosis and relevant drug delivery. The constant and continued search for small architecture and techniques enabled contact lens based sensors coupled with a drug delivery system. There has been a growing necessity to bring out traumatic free miniaturized sensors with a significant biocompatible drug delivery system, easily implantable into contact lenses for continuous monitoring of different physiological variables in the pursuit of timely intervention and beneficial results.

Though hard lenses or rigid gas permeable (RGP) lenses are known for maintaining their shape on the eye and high oxygen permeability, their susceptibility to scratches, displacement from the center of the eye, and prolonged wearing for adoption prompt users to choose soft contact lenses. Soft contact lenses with pronounced flexibility provide a more comfortable wear than hard lenses and they have high water retention capabilities, which enhances the oxygen flow to the cornea.^10,11^ The aforementioned features of soft contact lenses allows them to be used for different applications beyond vision correction, including color vision management.^12^ Interestingly microfluidic contact lenses have the advantage of being manipulated with liquids in picoliter precision; custom designed channels and reservoirs can help in colorimetric sensing and sustained drug release.^13,14^ So contact lenses can also act as a suitable drug delivery system in addition to being a diagnostic platform. Drug-laden soft contact lenses have gained importance in recent years since they establish a close contact to the cornea where the drug is to be administered. In terms of the residence time of the drug on the eye, contact lens based drug delivery can have extended effective residence time of over 30 min, as opposed to eye drops with hardly 2 min of residence time. In this regard contact lenses as a drug delivery system promise better bioavailability of the drug on the cornea.^15^ Since contact lens is a unique platform for biosensing and drug supply, it has an easy interface with the cornea, guaranteeing comfort^16^ and being safe from allergies^17,18^ and fungal attack.^19,20^

The aim of this review is to present an account of the chronological developments of contact lens sensors for ocular diagnosis. Furthermore, the use of contact lenses in glucose and IOP sensing along with their efficacy in drug delivery systems is studied thoroughly. This review comprehensively discusses the design of the sensors, the sensing mechanism, periocular implants, and modes of drug delivery. Finally, it is concluded with remarks on the potential technologies in ocular diagnosis and personalized medicine and future.

## Clinical Significance of Ocular Diagnostics

The power of accommodation by the eyes can sharply focus objects irrespective of the distance. The light-sensitive rod- and cone-shaped cells function as photoreceptors to visualize and differentiate colors and are sensitive to the intensity of light.^21^ Vision defects, such as hyperopia or farsightedness, myopia or nearsightedness, and presbyopia and astigmatism occurring due to lack of or loss of power of accommodation, shifting the focal point and the said vision defects can be rectified by using convex lens, concave lens, and the combination of both, respectively.^22−25^ Contact lenses emerged not only as a perfect replacement to lenses in vision correction, offering the wearers both comfort and convenience, but also a platform for point-of care diagnostics and drug delivery. Diabetes mellitus and glaucoma are some of the serious retinal disorders that can cause severe damage to optic nerves and eventual irreversible loss of eyesight in the long run, if prompt attention is not paid. Glaucoma, a silent cause of blindness, is caused by the impairment in the drainage system, resulting in an eventual accumulation of undrained fluid which causes elevation of IOP. The dormant nature of glaucoma compels continuous monitoring and lifetime medical support to shun further vision loss. All of the diabetic patients with uncontrolled blood sugar levels are vulnerable to glaucoma, cataract, and diabetic retinopathy of various degrees, which accounts for 1% of the blindness. Since these disorders cause catastrophic damage to vision, they demand early alert with timely diagnostics and medication.^26−29^ For a healthy person, the fasting glucose concentration should read between 70 and 140 mg dL^–1^ (3.9–7.8 mmol L^–1^) and postprandial plasma glucose concentration recorded should be around 200 mg dL^–1^ (11.1 mmol L^–1^) and anything exceeding 240 mg dL^–1^ (13.3 mmol L^–1^) demands medical attention;^30^ however in the case of tear fluid, the glucose concentration of 6 mg/(100 mL) (0.2 mmol L^–1^) accounts for a healthy condition and a concentration of 16.6 mg/(100 mL) (0.92 mmol L^–1^) indicates diabetes.^31^ Intraocular pressure is treated as one of the indicators of glaucoma; an elevated IOP reading of above 22 mmHg (normal is 12–22 mmHg) is known as ocular hypertension that can significantly point out glaucoma. The tear films consist of a mixture of proteins, neuropeptides, enzymes, and protective proteins, in addition to carbohydrates lipids and salts.^32^ Hence investigating the proteins from tear fluids can serve as biomarkers for human diseases.^33^ Proteomic study of tear fluid is an apt tool to diagnose several diseases even though the volume of tear fluid available is very low (<5 mL).^32,34^ Proteomic pattern variations in tears are the potential biomarkers to determine the disease and can provide aid for further diagnostics and treatment. Moreover, the pharmacological agents used can also be evaluated using proteomic analysis of tear fluid.^32^ Therefore, analyzing tear fluid instead of blood for target analytes’ concentration is a promising window. Contact lenses primarily worn for vision improvement and cosmetic and aesthetic purposes^35^ if embedded with a sensing unit can serve as a non-invasive/minimally invasive platform to carry out continuous monitoring to offer point-of-care treatment for retinal disorders. The main bottleneck to analyze tear fluid is the scantiness of sample providing a low amount of protein for the analysis as compared to that of blood protein (67.54 ± 11.53 g L^–1^) and its high dynamic nature which necessitates highly sensitive detection methods.^36^

Ocular diseases are common, and the administration of drugs is mostly ineffective owing to the blood–ocular barrier. The main drawback associated with traditional treatments such as eye drops is that they are handicapped owing to poor bioavailability, and intraocular injections can have serious side effects.^37^ Furthermore, the blood–aqueous barrier and blood–retinal barrier are highly responsible for the prevention of drug absorption from the blood.^38,39^ On administering drugs or autacoids, the endothelial barrier of the vessels in the retina is insensitive, and on the contrary, the vessels of the iris respond to any pharmacological manipulation with enhanced permeability.^38^ So, smart contact lenses incorporated with a drug delivery system along with sensors can be a great boon to ocular healthcare.

Contact lens based ocular diagnostics has attracted significant attention as they can monitor physiological parameters by directly detecting the biomarkers available in body fluids. Incorporation of electronics on contact lenses is quite challenging since the system needs flexibility, stretchability, reliability upon repeated eye blinks, and optical transparency for clear vision.^40^ The safe operation of contact lens sensors can be further hindered on a live eye due to the low oxygen permeability as a result of using opaque electronic components on lens-shaped plastic substrates rather than on a hydrogel lens. In order to make a successful contact lens based^41−44^ diagnostic device, the materials that make up the device and the antenna should be stretchable, transparent, and naive to the human body. Such wearables in medicine are to be designed with the capability to monitor user’s activities constantly and intimately without disturbing the user’s movements.^45^ The materials chosen for biosensor operation on soft lenses should be nontoxic, transparent, stretchable, reliable upon repeated stretching, folding, and bending, and oxygen permeable.^40,46^

In the recent past contact lens based ocular diagnostics and treatment strategies have been demonstrated. Contact lenses can be divided into rigid and soft contact lenses. Although rigid lenses are economical and long lasting and exhibit resistance to deposit building as compared to soft lenses, the latter is advantageous with a shorter period for the wearer to become accustomed to wearing them and for adjustment in addition to supplying more oxygen to the eye when they are worn.^47^ Given the advantages of soft contact lenses, they are more preferred in developing contact lens based sensors for ocular diagnostics. Soft contact lenses are typically made of hydrogels such as silicone with materials like hydroxyethyl methacrylate (HEMA), pHEMA,^2^ poly(ethylene terephthalate) (PET), poly(dimethylsiloxane) (PDMS),^48,49^ polyacrylamides (pAAms), 2,3-dihydroxypropyl methacrylate, poly(vinyl alcohols) (PVAs), and their combinations.^50^ In designing such sensors and drug delivery units, the two most important aspects to be given priority are (1) identifying appropriate transduction elements that can generate and communicate signals in the presence of analyte and (2) the design of the matrix to accommodate such components with physiological compatibility while sensitivity, reversibility, response time, and shelf life are to be equally taken care of as well.^51^

## Contact Lenses in Ocular Diagnostics

With the rapid advancement in wearable technology^52,53^ and the advantage of miniaturized sensing devices, effortless self-contained diagnostics at point-of-care settings have become realizable. More so, the role of contact lenses in ocular diagnostics were perceived as an efficient manifestation to carry out minimally and or non-invasive continuous monitoring of physiological variables and drug delivery^54^ apart from vision correction. The diagnostic capability of the contact lens sensors could be strengthened to sense more than one analyte simultaneously as well as individually eliminating the possibility of interference.^55^ This multianalyte sensing technique *avails one the liberty to design the sensor for a particular combination of analytes on the basis of the individual′s need*.

## Glucose Sensing

Over the past few decades the number of people including children with diabetes mellitus has dramatically increased globally, especially the quick spread of type-2 diabetes in the younger generation including children, adolescents, and young adults. Diabetes mellitus is caused by metabolic dysregulation that leads to impaired glucose metabolism. Such abnormal glucose metabolism and insulin resistance challenges the conversion of sugar into energy. In the pursuit of managing diabetes mellitus, glucose monitoring is a valuable step to diagnose the level of glucose concentration in blood.^3,56^ Fluctuations in the blood glucose level and delayed diagnosis may cause diabetic ketoacidosis and subsequent seizure.^57^ Therefore, in the event of treating diabetics continuous monitoring is a must to get rid of the health risks and long-term complications specifically associated with diabetes such as nephropathy, retinopathy, neuropathy, cardiovascular diseases, limb amputation, and kidney failure.^3,58^ The above said morbidities can happen to anyone with diabetes irrespective of whether one is insulin dependent or not.^58^

The traditional technologies are mostly invasive and are limited to offering point sample information but not competent for continuous monitoring.^40^ The patients with chronic diabetes conditions, having multidose insulin supports daily, are compelled to perform multiple blood glucose concentration measurements per day using finger pricks.^59^ Moreover self-measurement methods such as a portable glucometer may supply inaccurate readings with errors (±15%) and blood-borne diseases.^34,50^ Patient’s poor compliance owing to pain and inconvenience force the adoption of subcutaneously inserted electrochemical sensors to suffice the requirement in terms of continuous monitoring.^60,61^ Although these sensors have the potential to supply real-time and extended readouts with insulin supply on demand,^62^ they fail to provide complete solution owing to calibration requirement,^63^ time lag due to signal shift, and periodical replacement of the sensor.^64^ Minimally invasive sensors are also not exempted from calibration protocol and not completely non-invasive.^3^ In terms of reliability, enzymatic assay technique is a satisfactory choice; nonetheless the damage caused by the highly reactive and toxic byproducts^65^ limit its usage. Many other sensors incorporated into tattoos and skin patches and other minimally invasive sensors with automated feedback loop connected to an insulin pump can provide all-in-one solution but they are all incomplete in one way or the other to comply with non-invasive continuous monitoring.^66^ This potentially informs us that the choice of sample should be other than blood, to materialize a truly non-invasive platform for continuous monitoring of glucose concentration.

The options can reach out to many body fluids such as urine, saliva, and tear fluid, but tear fluid becomes the best choice as it can be obtained easily and continuously compared to urine and resilient against dilution.^44,67−72^ On top of that, the intermittent nature of this platform does not promise a real continuous monitoring feature.^44^ Thus, tear fluid based gadgets would more likely gain attention with contact lens serving as a carrier. With the discovery of glucose in tear fluid,^73^ establishment of its higher concentration in the tear fluid of diabetic patients than in healthy individuals,^44,74,75^ swelling in many folds,^31^ and the correlation between blood glucose and tear glucose,^74,76^ has assured tear fluid as vital for diagnosis. The availability of extremely low glucose concentration in tears (0.1–0.6 mM) as compared to blood serum (4–6 mM) compels the sensor to have high accuracy, selectivity, and interference rejection to avoid false alarms from ambiguous readouts.^77^ The human lachrymal liquids are basal, reflex, and psychic tears. The corneal surface hydration is maintained by basal tears while reflex tear is produced by the transient receptor channels which serve as defensive tools against stimuli or irritants. Psychological happenings secrete psychic tears which have a higher concentration of several hormones other than the two types of tears. This signifies the need to carefully design the contact lenses to ensure wearing comfort as a key feature to thwart the production of both reflex and psychic tears, as their excess quantity may result in dilution associated fallacious results and affect the accuracy.^78^

### Electrochemical Glucose Sensing

**Figure 1:**
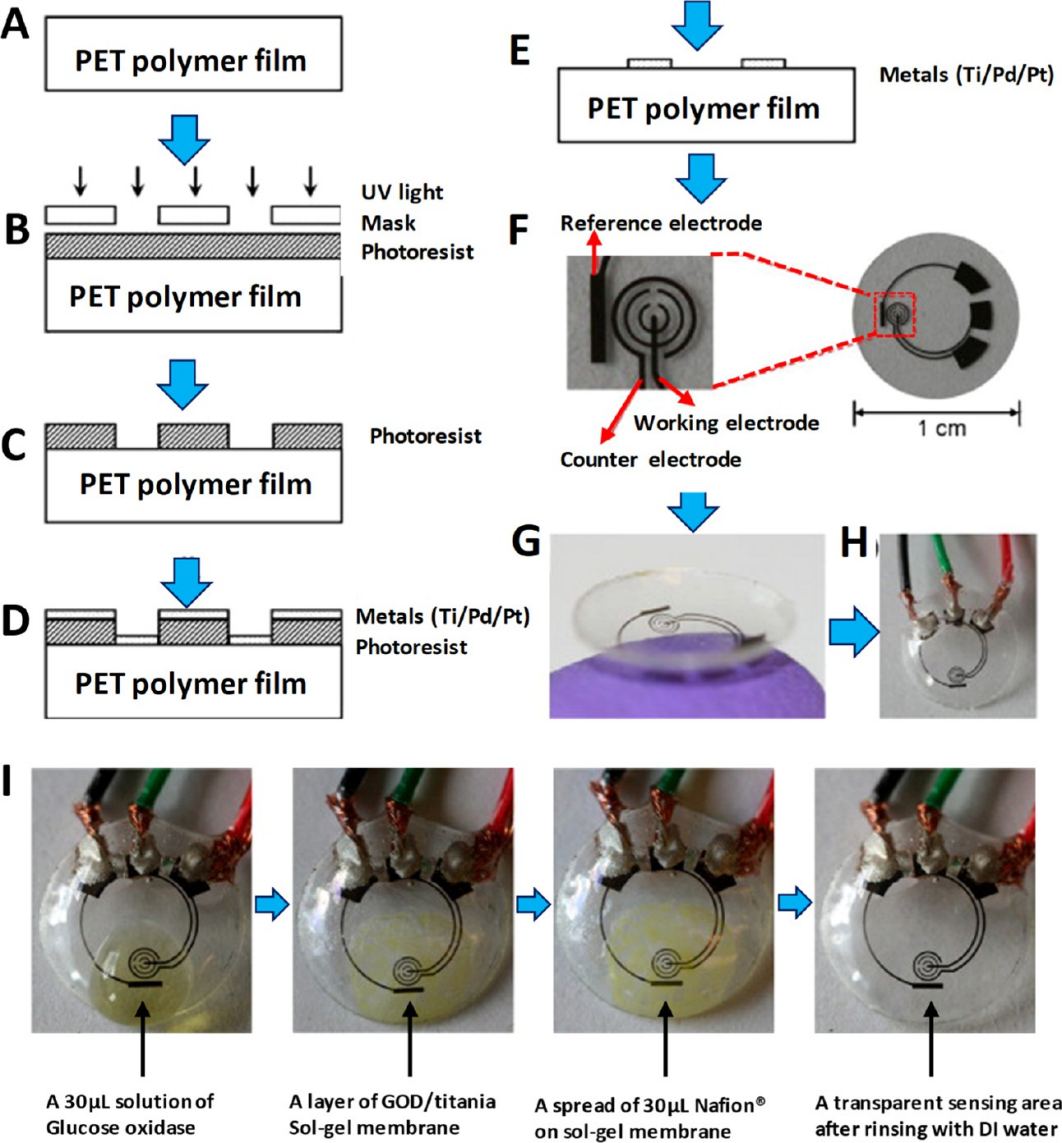
PET-based electrochemical glucose sensor fabrication process and results: (A) clean PET substrate prepared; (B) substrate covered by photoresist and exposed to ultraviolet (UV) light through a mask; (C) photoresist developed; (D) thin metal films evaporated on the sample; (E) after lift-off, metal pattern remaining on the surface (after this step, sensor cut out of polymer substrate and heat molded to the contact lens shape and functionalized with enzymes); (F) images of sensor after it has been cut out of the substrate; (G) image of completed sensor after molding held on a finger; (H) sensor hard-wired for testing: (I) sequential images of sensor as it goes through surface functionalization through pretreatment with GOD/titania/Nafion. Reprinted with permission from ref (44). Copyright 2011 Elsevier.

Electrochemical glucose sensors emerged to be a non-enzymatic method in glucose sensing, rectifying the disadvantages of enzymatic sensing. Ampherometic is an important electrochemical technique sensitive to analytes which measures current as a result of an electroactive material either losing (reduction) or gaining (reduction) an electron upon undergoing an electrochemical redox reaction.^79,80^ Yao *et al.*(44) introduced a tear fluid based contact lens glucose sensor, where a microstructured ampherometic glucose sensor is incorporated, on a transparent polyethylene terephthalate (PET) polymer substrate out of which the lens is formed (Figure 1). The glucose sensor was constructed by depositing three layers of metal (Ti, 10 nm; Pd, 10 nm; Pt, 100 nm) by evaporation on the polymer in succession. Working and counter electrodes have been formed as concentric rings to lower the resistance between the two electrodes. Immobilization of glucose oxidase (GOD) is achieved by titania sol–gel film to boost sensitivity, and interference rejection properties are improved with the help of Nafion. With a quick response of 20 s, high sensitivity of 240 μA cm^–2^ mM^–1^, and a minimum detection of <1 mM glucose, this sensor setup proved to be a robust one; however, it needs a wired readout, which can interrupt vision and cause inconvenience. It also lacks continuous monitoring and is not in a wearable form.

Iguchi *et al.*(81) developed a soft-micro electro mechanical system (MEMS) based flexible and wearable amperometric sensor to monitor glucose in tear fluid and tested it on the eyesight of a Japanese white rabbit. A flexible oxygen electrode was fabricated using MEMS technique with Pt working electrode and Ag/AgCl counter/reference electrode on which GOD was immobilized. The flexible oxygen electrodes consist of a polypropylene-based gas-permeable membrane of 25 μm thickness, an electrode setup (Pt (200 nm) and Ag/AgCl (300 nm) electrodes), a membrane filter, and a nonpermeable membrane of 50 μm. The electrolyte used was 0.1 M KCl (membrane filter), and the sensing work was carried out using a computer controlled potentiostat. The reaction occurring on the electrodes is given by the following eqs 1 and 2:

cathode (Pt):

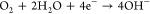
1anode (Ag/AgCl):

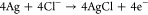
2

The rabbit was given oral administration of glucose (1 g of glucose per 1 kg of weight), and the tear glucose concentration was measured by attaching the sensing area on the pupil, and a controlled study was carried out (blood sugar level) using a monitoring kit. The polymer membrane based sensor showed a linear relationship between glucose concentration and the output current in a range of 0.025–1.475 mmol L^–1^, with a correlation coefficient of 0.998. The highest current was achieved at a pH of 7.0, and it was proportional to the change in temperature. The decrease in the current density beyond pH 7.0 and 45 °C is due to the degradation of the immobilized enzyme in alkaline medium and its thermal deactivation respectively marking pH 7.0 and 45 °C to ideal conditions for the enzyme to perform sensing. The tear glucose level showed a 3-fold increase (0.16–0.46 mmol L^–1^), and blood glucose level showed a 2-fold increase (3.7–7.6 mmol L^–1^). However, after glucose administration there is a 10–20 min additional delay in the change of tear glucose level as compared to the blood glucose level.

**Figure 2:**
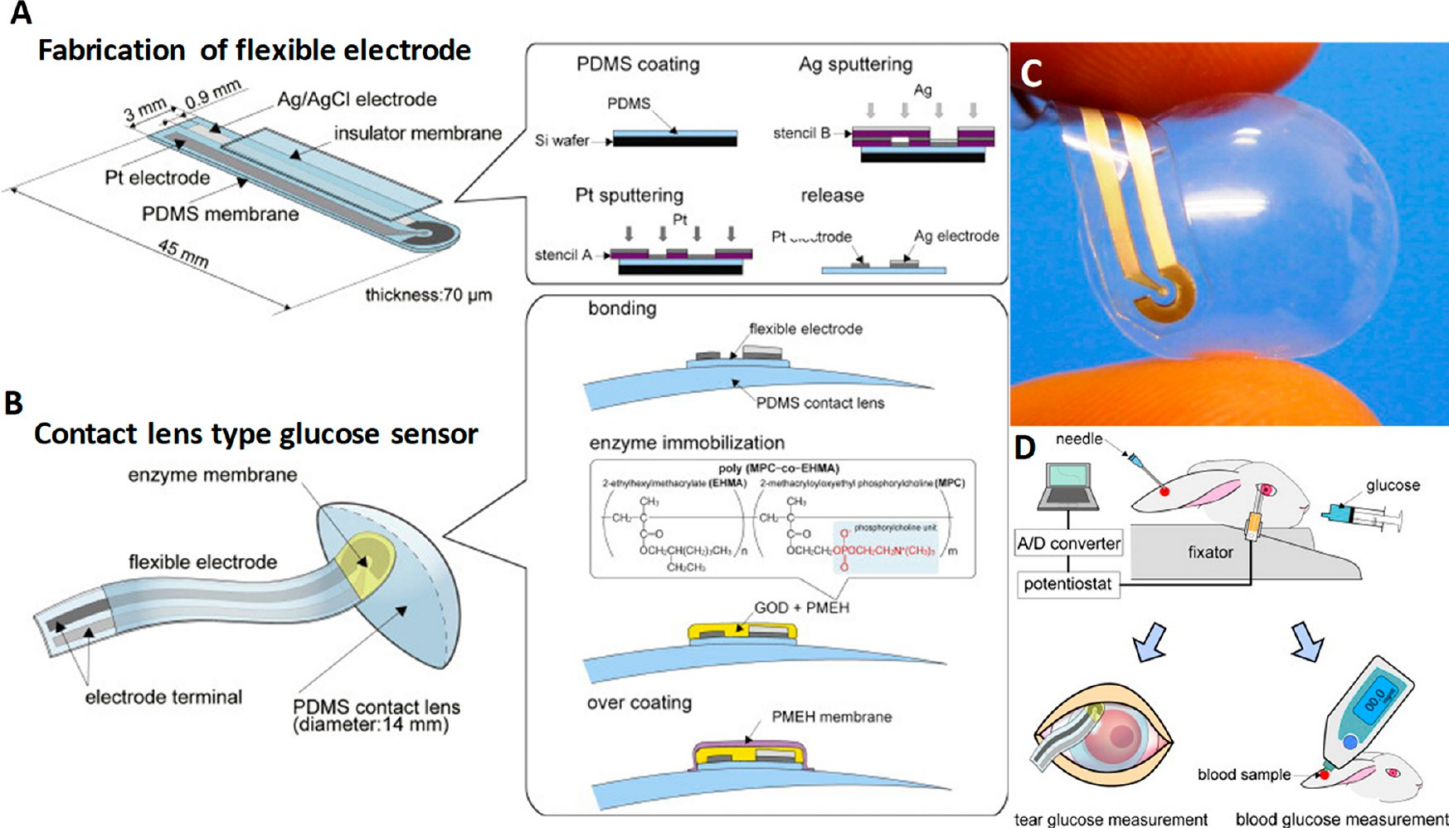
Composition and working principle of MEMS-based flexible wearable contact lens glucose sensor. Schemes of (A) formation of the flexible electrode on a 70 μm thick polydimethylsiloxane (PDMS) membrane and (B) flexible electrodes bonded onto the surface of the PDMS contact lens using PDMS and then GOD immobilized using PMEH onto the sensing region of the electrodes. Finally, enzyme membrane overcoated by PMEH: (C) digital image of flexible sensor; (D) measurement method of glucose concentrations in tear fluids using the flexible glucose sensor and a comparative measurement carried out simultaneously by a commercial kit. Reprinted with permission from ref (82). Copyright 2011 Elsevier.

The same group also reported^82^ yet another MEMS-based flexible contact lens biosensor using biocompatible 2-methacryloyloxyethyl phosphorylcholine (MPC) polymer and polydimethylsiloxane (PDMS) as sensing materials (Figure 2A–D), while the sputtered Pt and Ag (Ag/AgCl) served as working and reference electrodes, respectively. With a quick response this sensor established an appreciable linear relationship between glucose concentration and the output voltage in a range of 0.03–5.0 mmol L^–1^, along with a correlation coefficient of 0.999. This wearable sensor was also tested successfully on the eye site of a rabbit for tear glucose concentration and continuous monitoring of tear dynamics. Regardless of the advantage of device flexibility and accuracy, continuous monitoring can still be hindered since prolonged contact of the device with the cornea can induce eye irritation. Moreover, the need for a wired readout system and the influence of components such as GOD and H_2_O_2_^83−85^ pose a significant challenge for widespread adoption and commercialization.

### Fluorescent Probe-Based Glucose Sensing

**Figure 3:**
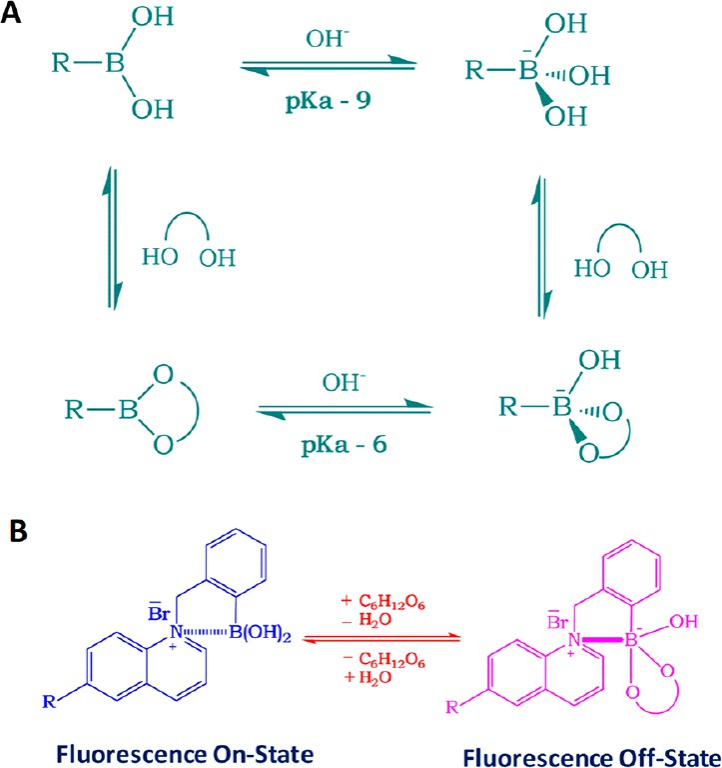
Boronic acid based glucose sensor and the sensing mechanism: (A) equilibrium for the boronic acid/diol (sugar) interaction; (B) schematic representation of the sensing mechanism for the charge neutralization mechanism with regard to glucose sensing.

The concept of incorporating glucose-sensitive, quinolinium backbone based fluorescent probes with contact lenses to detect tear glucose was achieved by Badugu *et al.*(86) Sufficient care was taken to obtain the fluorescent probes with lower p*K*_a_ values to ensure enhanced sensitivity toward physiological glucose in the acidic pH contact lens. Boronic acids have been known for their affinity to bind with diols,^87^ especially monosaccharides, and the idea of employing boronic acids for glucose sensing was sparked from the explorative research carried out by Yoon and Czarnik^88^ and Shinkai *et al.*(89) Boronic acids are basically weak acids with an sp^2^ hybridized boron atom and trigonal planar geometry. The electron deficient boron atom and two hydroxyl groups in boronic acid are capable of reacting with hard and strong bases such as OH^–^ to yield the anionic boronate form of sp^3^ hybridization and tetragonal conformation, with a high p*K*_a_ value of 9 (Figure 3A).^65,90^

Boronic acids (BAs) bind with diols through a dehydration process *via* reversible covalent bonds^65^ forming a boronic acid diester group which is more acidic (p*K*_a_ ≈ 6) than the boronic acid group because of a more electrophilic boron atom.^87^ The affinity of monophenylboronic acid group decreases from d-galactose to d-glucose; however, it exhibited greater affinity toward d-fructose. The substitution on the phenyl and/or the aromatic species where the boronic acid group is present, along with the molecular geometry, determines the affinity (*K*_D_) and the selectivity of the boronic acid group toward monosaccharides. This in turn can dictate the feasibility of employing boronic acid groups for sensing glucose. On the basis of this, glucose-sensitive probes can be prepared in mM and μM range for blood glucose^91−93^ and for tear glucose, respectively. Badugu *et al.*(94) developed a series of monosaccharide-sensitive fluorescent probes using boronic acid backbone. Then, they integrated them with disposable, off-the-shelf contact lenses and carried out glucose concentration characterization in the tear fluid. The different mechanisms underlying sensing are excited-state charge transfer (CT),^65^ charge neutralization,^51,95^ and photoinduced electron transfer (PIET).^96^ CT mechanism is applicable to fluorophores which have both boronic acid group and an electron donor group on them. The electron withdrawing BA group [-B(OH)_2_] binds with monosaccharide at suitable pH to form its excited anionic form ([-B(OH)(sugar)]-). In the process of charge transfer it loses its electron withdrawing nature, bringing change in its hybridization and fluorescence spectrum. The probes developed on the basis of the above-said mechanism when used in contact lenses exhibit both wavelength shifts and intensity changes upon encountering glucose, supplying information about tear fluid glucose concentration.

When adding glucose, the change in hybridization from sp^2^ to sp^3^ accompanied by geometrical change caused a fluorescence spectral change of the probe as a result of increased electron density on the boron atom. This, in turn, partially neutralized the positively charged quaternary nitrogen of the quinolinium moiety, termed as “charge neutralization–stabilization mechanism” (Figure 3B).^75,97^ Boronic acid groups induce change in fluorescence intensity in quaternary nitrogen containing compounds such as *N*-benzyl-6-methoxyquinolinium bromide [BMOQ] and *N*-benzyl-6-methylquinolinium bromide [BMQ] as the emission spectra of *N*-(boronobenzyl)-6-methoxyquinolinium bromide (*o*-BMOQBA) and *N*-(boronobenzyl)-6-methylquinolinium bromide (*o*-BMQBA) exhibited a steady-state decrease in the fluorescence intensity when the pH increased from 3 to 11. The quaternary nitrogen present in the BMOQ and BMQ interacts with the boronic acid group not only to reduce the p*K*_a_ values of *o*-BMOQBA and *o*-BMQBA probes that boost the probe’s sensitivity toward sugar but also to function as a stabilizer for the boronatediester complex. The contact lens sensors using *o*-BMOQBA and *o*-BMQBA as probes for glucose sensing^98^ showed a good response for the increase in glucose concentration while the latter exhibited greater response to the analyte than the former. This probe-based contact lens is reported to have 90% response time in 10 min, but successful usage requires an excitation and detection device which can be complemented to the contact lens that undergoes glucose concentration based color change.

March *et al.*(99) introduced the concept of non-invasive glucose monitoring of the aqueous humor using a contact lens and paved the way to practical applications.^100^ The contact lens was fabricated by incorporating a couple of fluorescent indicators in the polymer matrix. The indicators are bound to each other in the absence of glucose, and when they come in contact with glucose, they dissociate so that fluorescence is detected and the outcoming signal can be read by a recording unit combined with an illumination placed in front of the eye.^69,100^ They developed a holographic contact lens and succeeded with clinical trial based on reversible chelation of diols such as glucose to boronic acid derivatives.^101^ The spectral effects shown by hologram grating resulting from the volumetric change is termed as “Denisyuk hologram” based on Lippmann color photography which is different from embossed holograms. The hologram patterns are created with the aid of laser light reflected from a mirror producing a classical standard wave which is recorded on the polymer matrix. The spacing between the fringes expands diffracting longer wavelength when the polymer matrix undergoes a bulge in its volume. 3-Acrylamidophenyl boronic acid is the probe capable of chelating with glucose as reversible binding ligand. The sensor was made by embedding 3-acrylamidophenyl boronic acid in acrylamide copolymer hydrogel matrix and incorporated on to a contact lens made of Nelfilcon A. The boronic acid based ligands used as glucose probes do have a recorded hologram within them that make the holographic glucose sensor.^102−104^ The silver halide fringes present in the holograms function as sensitive wavelength filters reflecting a narrow band of frequencies on the basis of the spacing between the fringes when the hologram is illuminated, and fringe separation on the basis of the polymer bulging selectively reflects the light of a particular wavelength. The expansion of interference fringes when the ligand binds with glucose by reversible covalent bonds, causes color change of the light coming off of the hologram which is used to quantify the glucose concentration present in the tear fluid. An *in vivo* clinical trial was carried out successfully for a period of 1.5 h. The major drawback in using boronic acid for glucose sensing is that they are not specific unlike ligand assisted fluorescent methods. While sensing glucose, there is a possibility of fructose and other diols to interrupt, recording erroneous readings and yielding ambiguous results.^101^

### Photonic Crystal-Based Glucose Sensing

**Figure 4:**
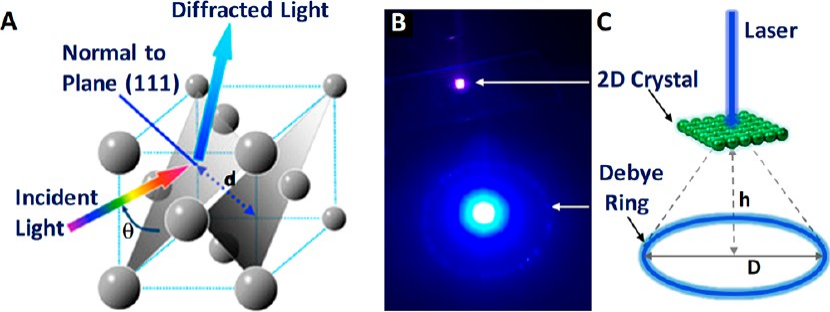
(A) Schematic illustration of GCCA’s diffraction phenomenon from (111) planes of crystalline colloidal array (CCA) with a FCC arrangement that follows Bragg’s law. Reproduced with permission from ref (109). Copyright 2014 MDPI. Debye diffraction ring measurement: (B) digital image of Debye diffraction ring resulting from a 2D gelated monolayered colloidal crystal (2D GMCC; under 445 nm wavelength laser light); (C) schematic representation of the principle for Debye diffraction ring detection. Reprinted with permission from ref (85). Copyright 2018 American Chemical Society.

Photonic crystals typically have a periodic refractive index leading to an optical band gap that controls the flow of light of a certain frequency range. Two-dimensional (2D) photonic crystalline arrays (CCAs) serve as photonic crystal materials and sensors^105^ due to their appreciable response to external stimuli and visual diffraction.^106^ Well-ordered 2D assemblies can be achieved using dip coating, spin coating, and electrophoretic deposition of colloidal particles on the substrates.^105^ These highly ordered photonic crystals (PCs), with their periodicity in their refractive index on the order of the wavelength of light, diffract the visible light in accordance to array spacing obeying Bragg’s law (Figure 4A).^107,108^

Such stimulus-responsive hydrogels incorporated into 3D PC structures through gelation show a volume change and red shifting of the diffracted light as a response to a stimulus such as glucose. The volume change and the subsequent change in the wavelength of the diffracted light are associated with the change in the lattice constant of the crystalline colloidal array (Figure 4B,C).^110^ Chemical sensors are designed in such a way to respond to the target analyte and produce a visually distinguishable color change with a change in optical properties (diffraction) as a response to a chemical signal. The selective binding of the molecular recognition agent to the analyte elevates the osmotic pressure and subsequent volumetric change in the hydrogel.^111^ In the case of glucose-sensitive hydrogels (GSHs) when bound to glucose, they undergo a volumetric change in proportion to glucose concentration because of the formation of reversible cross-links accompanying a change in Debye diffraction ring diameter with the increase in the mean separation between the photonic crystals.^106^ This principle of volume expansion and shrinkage in stimulus-responsive GSH opens another window for glucose sensor research. Alexeev *et al.*(112) developed a glucose-sensitive photonic crystal by fabrication of a crystalline colloidal array fixed in a polymeric network of a polyacrylamide–poly(ethylene glycol) hydrogel with pendent phenylboronic acid groups making a holographic hydrogel. This hydrogel material is incorporated with a contact lens, and upon illuminating the hydrogel, the variation in the wavelength of the refracted light reflects the concentration of glucose. The patient can infer the glucose level by matching the color of the patch to the reference.^65,110^ Polymerized crystalline colloidal array (PCCA) is a photonic crystal material, made of embedded crystalline colloidal array in a polymer hydrogel network with molecular recognition element. When the analyte (glucose) comes in contact with the molecular recognition element present in the PCCA-based sensor, the hydrogel undergoes change in its volume, which in turn red shifts the light diffracted by the CCA of the polystyrene particles. They employed fluorinated boronic acid derivative such as 4-amino-3-fluorophenylboronic acid (AFBA; p*K*_a_ = 7.8)^113^ as the molecular recognition elements and compared its performance with that of 4-carboxy-3-fluorophenylboronic acid (CFBA). These boric acid derivatives have lower p*K*_a_ values than any aromatic ring substituted boric acid derivatives, having an advantage in glucose sensing in terms of improved sensitivity as a decrease in p*K*_a_ values of the molecular recognition elements enhances pH dependent glucose response in the physiological pH range. When light is incident on the PCCA of polystyrene particles it refracts the light of a wavelength with a red shift due to the volumetric swelling of the hydrogel, arising from the interaction of the analyte with the molecular recognition element. This red shift of the diffracted light corresponds to the concentration of glucose at physiologic ionic strengths and at pH 7.4. Therefore, the glucose concentration can be determined from the diffracted band shifts. The volume changes of hydrogel caused when glucose chelates to the boron derivatives *via* bis-bidendate cross-linking. AFBA-AA-PEG PCCAs (AA, acrylamide; PEG, poly(ethylene glycol)) composition used for sensing red shifts of the diffracted light beyond the 20 mmol L^–1^ concentration of glucose and the color changes that were in accordance with an increased glucose concentration are significant. The reported material (AFBA-AA-PEGPCCAs) could sense the glucose in the range of 100 μmol L^–1^ in the tear fluid and detection limit of 1 μmol L^–1^ in synthetic tear fluid. Although this method is successful in the fabrication of diagnostic contact lenses to monitor glucose concentration, the results are validated only by the patients visually without the help of any instrumentation offering a standard reference.

**Figure 5:**
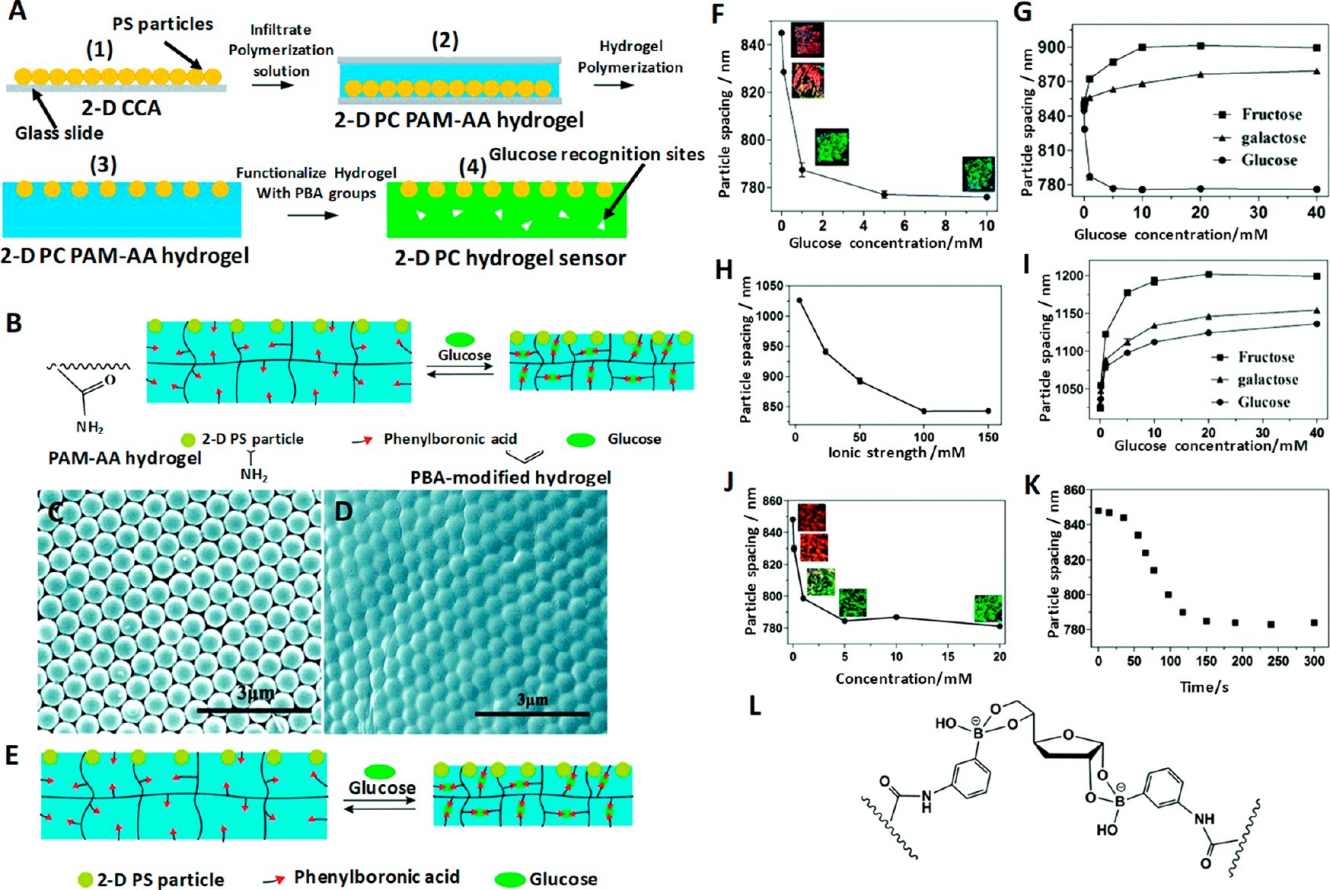
PBA modified hydrogel glucose sensor fabrication, sensing mechanism, and results. (A) Fabrication of a glucose-responsive 2D PC PAM-AA hydrogel: (1) 2D CCA on a glass slide; (2) infiltration of prepolymerization solution into the 2D CCA and initiation of the polymerization by UV light; (3) separation of the 2D PC PAM-AA hydrogel film from the glass slide and washing it with water; (4) functionalization of the 2D PC PAM-AA hydrogel with PBA groups. (B) Chemistry of coupling PBA recognition groups to the hydrogel matrix. (C) SEM image of PS 2D CCA on a glass slide. (D) Surface of the 2D PC PAM-AA hydrogel with the PS 2D CCA monolayer embedded. (E) Scheme of the shrinking response of the 2D PC PAM-AA hydrogel response to glucose. (F) Dependence of the particle spacing of the 2D PC PAM-AA hydrogel on glucose concentration in CHES buffer (ionic strength, 150 mM; pH 9).The inset shows the diffraction color of the 2D PC hydrogels. (G) Comparison of the 2D PC hydrogel responses to glucose, fructose, and galactose in CHES buffer (10 mM, pH 9). (H) Ionic strength dependence of the 2D PC PAM-AA hydrogel in CHES buffer (pH 9). (I) Comparison of the 2D PC PAM-AA hydrogel responses to glucose, fructose, and galactose in low ionic strength buffer (10 mM, pH 9). (J) Glucose concentration dependence of the 2D PC PAM-AA hydrogel in a pH adjusted tear fluid. (Inset) Diffraction color changes from red to green with increasing glucose concentration. (K) Kinetics of glucose sensing of the 2D PC PAM-AA hydrogel for 10 mM glucose in an artificial tear fluid (pH was adjusted to pH 9). (L) Bis-bidentate glucose–boronate complexation with the furanose form of glucose. Reprinted with permission from ref (106). Copyright 2014 The Royal Society of Chemistry.

Despite the advantages of 3D colloidal arrays in glucose sensing, it is still not a perfect solution due to the difficulty in obtaining an array of good order, poor selectivity, and onerous self-assembly procedure. Self-assembly of 2D colloid arrays such as monodispersed PS particles on an air–water interface are simpler and less time-consuming. The preparation of the 3D arrays is not only tedious but also involves sophisticated steps such as dialyses and etching, making 2D CCA more preferred over 3D-CCA. 2D CCA that is prepared at the air–water interface is both easy and rapid to form. A self-assembled 2D CCA monolayer exhibited advantages over 3D CCA and was better incorporated into hydrogels.^106^ Xue *et al.*(106) reported a new 2D PC hydrogel based glucose sensor, whereby they incorporated a polystyrene-based 2D CCA monolayer on a phenylboronic acid (PBA) modified hydrogel with high sensitivity to glucose over other sugars. The typical sensor is assembled by embedding the 2D CCA of PS particles on poly(acrylamide-*co*-acrylic acid) (PAM-AA) hydrogels followed by chemically modifying 2D PC PAM-AA hydrogel with PBA derivatives which play the role of glucose binding sites as shown in Figure 5A–L.

With such a sensor setup, glucose sensing is carried out by visual readouts, specifically by measuring the Debye diffracted ring diameter originating as a result of volumetric change of the microstructure when bound to glucose. The Debye diffraction of the microstructure obeys
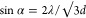
3where α is the forward diffraction angle of the Debye diffraction, λ is the incident wavelength, *d* is the nearest neighboring particle spacing, and α can be calculated by
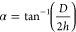
4where *h* is the distance between the 2D CCA and the screen and *D* is the Debye ring diameter, and from this the 2D CCA particle spacing associated with the volumetric change of the hydrogel can be calculated. This method can be carried out with the help of a laser pointer, in a dark room, to evade the intrusion of light from the surrounding and the visible spectrometer is not used as it cannot measure the diffraction wavelength if the wavelength falls beyond the measurable angles. With increasing the glucose concentration, the diffraction color of the sensor is blue-shifted. This method relies on visual detection and has the accuracy to detect glucose concentrations as low as 0.1 mM.

**Figure 6:**
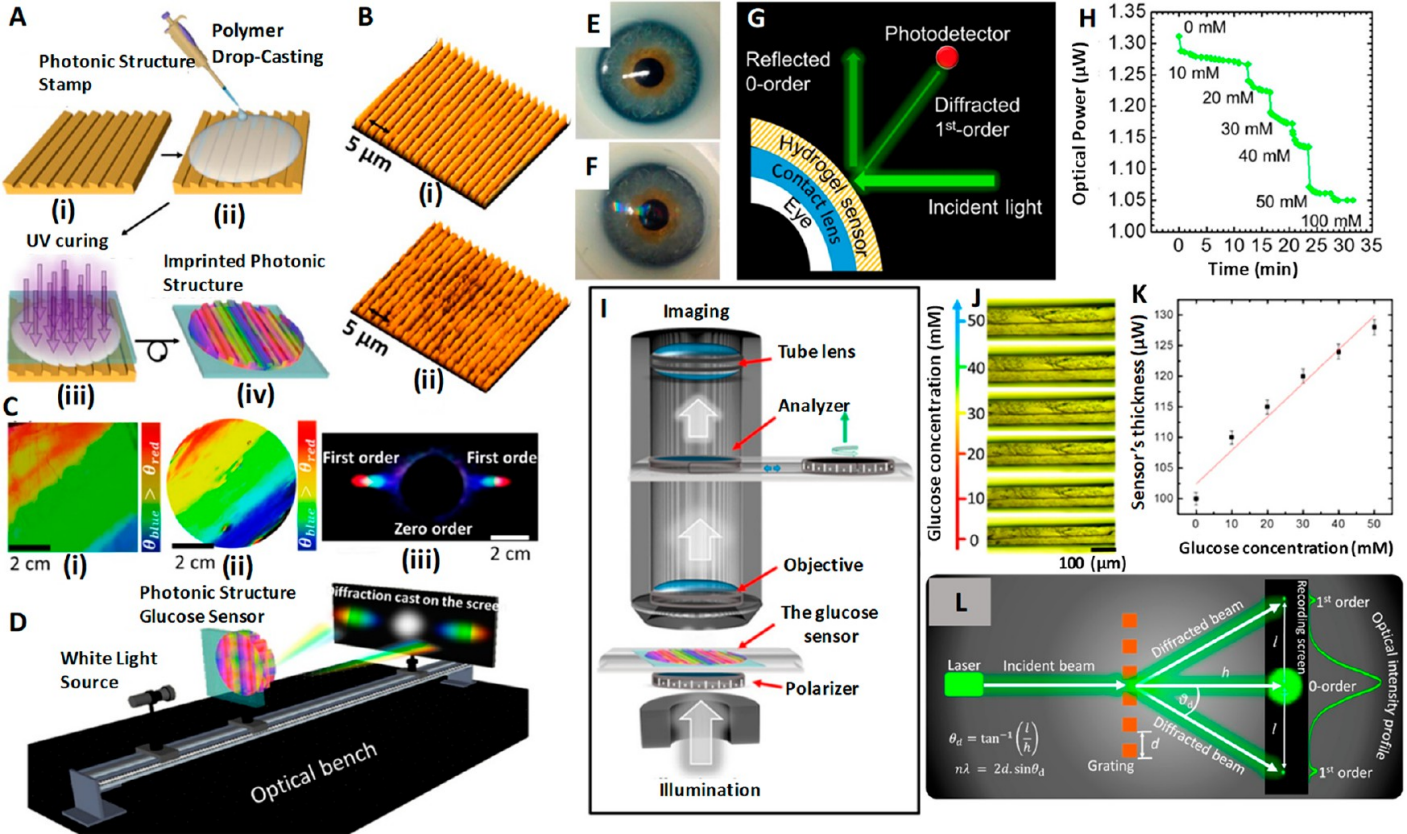
1D PS embedded contact lens sensor for tear glucose measurement, fabrication process, working principle, measurement protocol, and test results. (A) Schematic illustration of the fabrication process of the one-dimensional photonic structure based hydrogel glucose sensor: (i) PS master based stamp; (ii) drop casting of PS along with monomer solution; (iii) UV enhanced photo-polymerization of monomer solution; (iv) replica of the stamp peeled off from the master PS. (B) Optical microscope images of (i) the master PS and (ii) the stamped responsive hydrogel. (C) Photographs of (i) the original grating, (ii) the prepared hydrogel sensor, and (iii) the diffraction pattern (transmission) for the white light source by the PS sensor. (D) Schematic of the setup used to project transmitted diffraction patterns. (E) Photograph of a commercial contact lens on an artificial eye. (F) Photograph of the sensor attached to the contact lens and placed on the eye model. (G) Schematic diagram of the measurement setup. (H) Reflected optical power of the diffracted first order for various glucose concentrations (0–50 mM) *vs* time measured using the optical power meter. (I) Schematic for the setup of measuring the transmission of the sensor under various polarization angles. (J) Microscopic images of the 1D PS sensor’s cross-section in various glucose concentrations. (K) Change in the sensor’s cross-section as a function of glucose concentration. The scale bars show standard error (*n* = 3). (L) Schematic setup for recording the diffraction in transmission mode. Reprinted with permission from ref (3). Copyright 2018 American Chemical Society.

Chen *et al.*(85) assembled polystyrene into a two-dimensional template and combined it with 4-boronobenzaldehyde-functionalized poly(vinyl alcohol), a glucose-sensitive hydrogel. The above sensing unit was attached to a contact lens and successfully determined the glucose concentration in the ranges of 0–20 mmol^85^ and 0–50 mmol^109^ within 180 s. Elsherif *et al.*(3) applied the glucose sensing principle by photonic materials and developed a photonic microstructure based sensor that is capable of offering point-of-care continuous glucose monitoring using mobile phones. The wearable contact lens glucose sensor was formed by printing a photonic microstructure with 1.6 μm periodicity on a GSH film functionalized with phenylboronic acid to establish a bonding with glucose, and this freestanding photonic structure (PS) sensor is incorporated into commercial lenses (Figure 6A–L). Phenylboronic acid is considered to be an artificial mimic of lectin since it is capable of forming cyclic esters by extending strong and reversible covalent bonding with 1,2- or 1,3-cis-diols like glucose forming a five- or six-membered boronic cyclic ester in aqueous media,^114^ providing a wider platform for glucose sensing. When the microstructure binds with glucose, the former shows an expansion in its volume, and by correlating the resulting periodicity constant with glucose concentration, the glucose level in the tears can be continuously measured and monitored using a smartphone. The induced volume difference in glucose concentration is reflected in Bragg peak shifts since the dielectric photonic crystal (PC) array diffracts light of a particular wavelength selectively, conforming to the Bragg’s law. The difference in volume caused by glucose concentration is quantified using the periodicity constant and the diffraction efficiency/the power of the first-order spot for the freestanding PS sensor and the contact lens based sensor, respectively. This sensor proved to be efficient with high sensitivity, a quick response time of 3 s, and a short saturation time of 4 min within the physiological conditions of pH 7.4 and 150 mM ionic strength, thus promising a sophisticated glucose sensing platform at home settings.^3^ The same group also achieved contact lens aided glucose sensing using laser written micro-imprinted optical diffuser pattern on phenylboronic acid functionalized hydrogel.^115^ The said methods to measure glucose concentration using smartphone definitely offer a higher pedestal for a truly non-invasive and continuous self-monitoring.

### Graphen-Based Glucose Sensing

Kim *et al.*(40) developed a graphene oxide (GO) based multifunctional ocular contact lens sensor to monitor both glucose level in tear fluid and intraocular pressure simultaneously and individually by recording different electrical responses, making use of the resistance and capacitance of the electronic device. A hybrid structure made of graphene and silver nanowire (AgNW) is used as the key component since it has well-pronounced transparency (>91%), stretchability (∼25%), very low sheet resistance compared to the individual components (graphene and AgNW), and negligible transconductance, which all fit the requirements. The field-effect transistor based sensor is formed on a Si wafer with a 300 nm thick SiO_2_ layer where the hybrid serves as transparent and stretchable source/drain (S/D) with graphene as a channel. Furthermore, graphene–AgNW hybrid stands out as a promising candidate for soft contact lens based wearable electronics as it exhibits almost a constant resistance (Δ*R* < 6%) for 5000 cycles of stretching and relaxation. Parylene is the preferred substrate owing to its superior mechanical properties such as stretchability, strength, and intraocular biocompatibility.^116^

**Figure 7:**
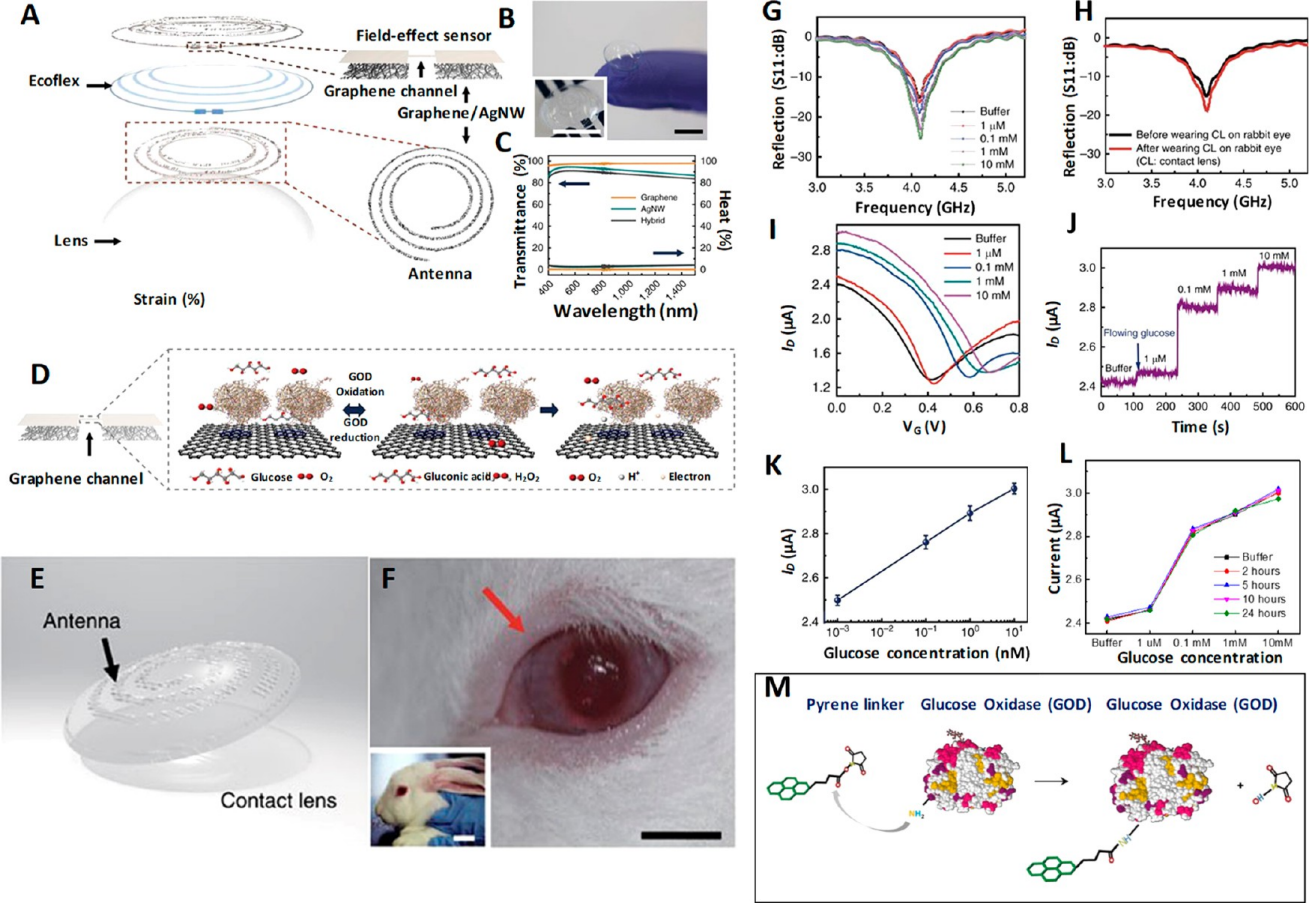
Graphene/AgNW hybrid based field-effect sensor for IOP measurement. Design, sensing mechanism, *in vivo* testing, and results. (A) Schematic of the GO/AgNWs-based wearable contact lens sensor, integrating the glucose sensor and intraocular pressure sensor. (B) Photograph of the contact lens sensor. Scale bar, 1 cm. (Inset: Close-up image of the antenna on the contact lens. Scale bar, 1 cm). (C) Optical transmittance and haze spectra of the bare graphene, AgNWs film, and their hybrid structures. (D) Schematic illustration and principle of glucose detection with the GOD–pyrene functionalized graphene. (E) Schematic illustration of the transparent glucose sensor on a contact lens. (F) Photographs of a wireless sensor integrated onto the eyes of a live rabbit. Black and white scale bars, 1 and 5 cm, respectively. (G) Wireless monitoring of glucose concentrations from 1 to 10 mM. (H) Wireless sensing curves of glucose concentration before and after a contact lens is worn on an eye of live rabbit. (I) Transfer (*I*_D_–*V*_G_) characteristics of the sensor at varied concentrations of glucose (*V*_D_ = 0.1 V). (J) Real-time continuous monitoring of glucose concentrations (*V*_G_ = 0 V). (K) Calibration curve generated by averaging current values and the glucose concentration from 1 to 10 mM. Each data point indicates the mean value for 10 samples, and error bars represent the s.d. (L) Stability of the glucose sensor: calibration currents for various glucose concentrations with the passage of time. (M) Amide bond between pyrene linker and glucose oxidase: formation of amide bond resulted with the nucleophilic substitution of *N*-hydroxysuccinimide by amine group on protein. Panels A–M reprinted with permission from ref (40). Copyright 2017 Springer Nature.

The hybrid sensing components are integrated into a resistance (*R*), inductance (*L*), and capacitance (*C*) circuit and operated wirelessly at a radiofrequency to enhance real-time *in vivo* glucose detection and *in vitro* monitoring of intraocular pressure on a rabbit eye and bovine eyeball, respectively. This sensor is highly sensitive to the glucose concentration in the tear fluid in the range of 0.1–0.6 mM and has a detection accuracy of 1 μM in the presence of ions and other interfering molecules in the tear fluid with a pronounced stability of 24 h. Immobilization of glucose oxidase on the graphene channel is achieved using pyrene molecules though π–π stacking, and the amide bond from nucleophilic substitution of *N*-hydroxysuccinimide aids the bonding to pyrene linker molecule as shown in Figure 7A–M. AgNW is protected from the tear fluid to avoid the formation of insoluble salts (AgCl) with chloride ions in the tear fluid which would otherwise harm the eyes. Similarly, a two-layer passivation is suggested to protect the sensor from tear fluid because the grain boundaries in graphene can affect the effectiveness of the seal if the lens is worn for a long time. The oxidation of glucose to gluconic acid and the reduction of water to hydrogen peroxide is catalyzed by GOD. The drain current which depends on the concentration of charge carriers in the channel is directly proportional to the concentration of glucose.^40,44,117,117^ All functions of this device from supplying power to sensing data are carried out wirelessly. This device has the advantage of monitoring both glucose and IOP; it also provides independent readouts which help in designing a contact lens sensor to perform multiple tasks. In the recent past ultrathin molybdenum disulfide (MoS_2_) transistors and gold (Au) wire components housed in contact lens sensors for the determination of glucose concentration along with corneal temperature have been reported. With profound flexibility, mechanical strength, wearing comfort, and biocompatibility they promise a multifunctional sensing platform in the future.^118^

### Microfluidics-Based Glucose Sensing

Microfluidic contact lenses belong to the family of soft contact classes made of hydrogels with microfluidic capabilities, namely, microcavities and microchannels.^119,120^ These contact lens sensors have several advantages over the conventional ones with electronic sensors by means of continuous fluid analysis,^4,121,122^ flexibility and direct detection of analytes with colorimetric,^8^ or liquid displacement techniques,^120^ thus providing high accuracy and reliability. The usage of tears and reagents in low volume, precisely in measures of picoliters, in leak proof microstructures renders high precision in sensing. The conductive liquids with high intrinsic deformity and physicochemical stability can ensure better outcomes than solid-state counter parts.^123−125^ The principle of sensing using microfluidic contact lenses lies on the design of microstructures and electronic functionalities with the success of the device depending on the liquids’ material characteristics.^49^ Microfluidic contact lenses of required dimensions and geometries are achieved by thermoforming,^49^ injection-molding,^54^ polymer casting,^126^ microlithography,^119^ and imprinting^127^ with suitable templates. The combination of laser patterning and embedded templating for the manufacture of microfluidic contact lenses can overcome the disadvantages of the conventional methods, and when customized, they can transcend soft contact lenses.^119^

Methacrylate poly(dodecanediol citrate) polymer (mPDC) is a UV-curable hydrophilic biopolymer. They are well-suited for contact lens applications because they have superior mechanical and biocompatibility characteristics. Yang *et al.*(120) fabricated a multitasking colorimetric microfluidic contact lens setup to measure the concentration of three analytes; (i) glucose, (ii) chloride, and (iii) urea, simultaneously and independently. The sensor setup is free from the conventional electronic structures and inductors so that vision interruption from building components is alleviated. Therefore, the colorimetric detection comes in handy to the wearer.

The contact lens sensor fabricated has microchannels engraved the inner contact lens adhering to the surface of the cornea, a colorimetric analysis unit planted in the cavity of the inner lens, and an outer lens meeting the eyelid that extends an open-loop microchannel and reservoirs along with an inner lens. A single unit of microchannel has an inlet, a detection zone, a reservoir, and an outlet; three such identical microchannels were embedded into the contact lens. When tear flowed into the microchannel, the embedded colorimetric analysis unit would show visible color changes by reacting with an analyte in the tear. By capturing the picture of the color change with a smartphone and comparing the RGB values, one can obtain information on the concentration of the analytes in the tear. Glucose is detected from the red color observed when peroxidase condenses phenol and colorless 4-aminoantipyrine.

**Figure 8:**
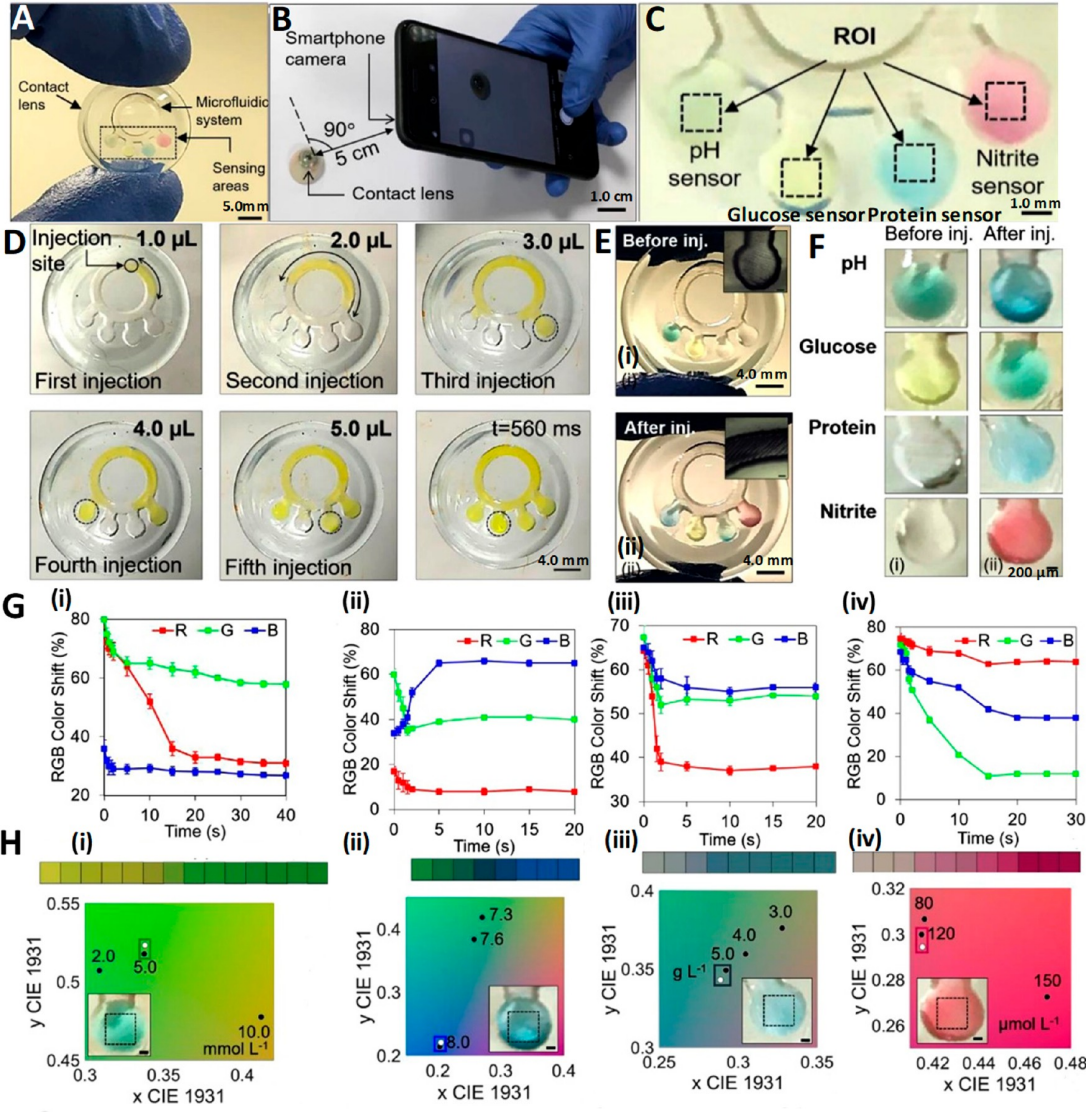
Multitarget sensing microfluidic contact lens, design, methods of fabrication, testing protocol, and diagnostic results. (A) Digital image of a contact lens sensing platform with multiple targets. (B) Color change of the sensors imaged using a smartphone camera. (C) Photographs of the sensors serving as inputs to the customized MATLAB algorithm, where the region of interest (ROI) can be selected. Characterization of microfluidic contact lenses: (D) Fluid flow characterization with fluorescein aqueous solution. Five consecutive injections amounting to 1 μL each were performed from the indicated injection site. Within 560 ms, the fluid reached all of the sensing site. (E) Characterization of contact lens sensors with artificial tear fluid. Photographs of a contact lens sensor before (i) and after (ii) artificial tear fluid injection. (F) Representation of smartphone readouts on contact lens sensors before (i) and after (ii) artificial tear fluid injection. (G) Red, green, and blue color shift over time for (i) glucose, (ii) pH, (iii) protein, and (iv) nitrite biochemical sensors. (H) CIE 1931 chromaticity diagrams obtained with the algorithm after inputting the imaged sensors. The algorithm allowed selection of the region of interest, indicated with black dotted lines. The corresponding normalized color is plotted in the chromaticity space calibrated with the points of the sensor of interest (white dots) and compared to the calibration values (black dots). The nearest calibration point gives the concentration readout. Readouts refer to (i) glucose, (ii) pH, (iii) protein, and (iv) nitrite sensors. Reprinted with permission from ref (4). Copyright 2020 Elsevier.

To bring advantages to the exiting devices Moreddu *et al.*(4,128) adopted CO_2_ laser ablation to bring out a microfluidic contact lens sensor capable of detecting multiple analytes simultaneously (pH, glucose, nitrite ions proteins, and l-ascorbic acid). The sensor was fabricated on a commercial contact by carving a ring-shaped microchannel bearing four branches with microcavities in which chromogenic compound sensors pertaining to the analytes were introduced, as shown in Figure 8A–F. The variation in color exhibited on the basis of the kind and concentration of the analaytes can be captured using a smartphone and the RGB values can be evaluated.

A two-step enzymatic method is used to detect glucose involving glucose oxidase/peroxidase (GOD/POD).^128^ Hydrogen peroxide obtained as byproduct when d-glucose is oxdized to d-gluconolactone oxidizes 3,3′,5,5′-tetramethylbenzidine (TMB). On the basis of the concentration of glucose (0–20 mmol L^–1^), the senor exhibits a color change ranging from yellow to green with varying intensities (Figure 8G(i),H(i)).^4^ Yellow-greenish color is indicative of healthy condition, while clear yellow and dark green colors correspond to down-regulated and up-regulated sugar levels. The reported sensor showed a sensitivity of 1.4 nm/(mmol L^–1^) of glucose and a limit of detection of 1.84 mmol L^–1^.

## Microfluidics-Based pH Sensing

The pH sensing is carried out using a mixture of methyl red (pH of 4.3–6.2), phenolphthalein (pH of 8.2–12.0), and bromothymol blue, a weak acid subjected to color change in alkaline media, to suit a range of pH both in acidic and basic windows. The sensor is boasted to have a rapid color saturation within 5 s and an excellent sensitivity of 12.23 nm/(pH unit). The colorimetric sensor can provide information on pH *via* color variation from yellow (mild acidic pH) to blue (alkaline pH due to Rosacea disease^129^) and green (healthy) (Figure 8G(ii),H(ii)).^4^ To monitor mild variation in ocular pH in the range of 6.5–7.5, anthocyanin-functionalized contact lens sensors (non-microfluidic) are used. On the basis of the color shift shown by anthocyanin with different concentrations of hydrogen ion, the information on ocular pH can be obtained (pH 6.5, pink; pH 7.0, purple; pH 7.5, blue).^9^

## Microfluidics-Based Protein Sensing

Diagnosis of certain disorders such as diabetic retinopathy,^130−132^ aniridia,^128,133^ keratoconus,^134^ and various dry eye diseases^135^ can be easily detected by analyzing tear proteomics. The protein concentration in tears ranges from 3 to 7 μg μL^–1^, and keratoconic tears have almost 50% (3.86 mg mL^–1^) of the level of proteins present in healthy subjects. Moreover, a drop in the amount of individual proteins such as secretory immunoglobulin A (IgA) and lactoferrin is possible too. Therefore, diagnosis of tear protein level is extremely important for the treatment of keratoconus as it can result in bulging of cornea into a conical shape.^122,134^ The principle of protein sensor relies on the color change in the reflected light when 3′,3′,5′,5′-tetrachlorphenol-3,4,5,6-tetrabromsulfophthalein reacts with hydrogen ion of the amino acid producing an anode of the same compound.^128^ For the protein concentration in the range of 0.5–5.0 g L^–1^, the color change is observed from beige to light blue with the sensitivity of 0.49 nm/(g L^–1^) for proteins and a LOD of 0.63 g L^–1^ (Figure 8G(iii),H(iii)).^4^ The sensor always displays blue color regardless of the proteins concentration in tears; however, for a healthy subject with more than 5 g L^–1^ protein intense blue color can be observed and low-intensity is ascribed to reduced protein level associated with keratoconus (3 g L^–1^).^134^ Urea being the end product of protein decomposition, determination of its concentration level with the help of Jung method involving a chromogenic agent is vital to monitor health.^120^

## Microfluidics-Based Nitrite, Chloride, and Sodium Ion Sensing

Determination of nitrite ion concentration in tear fluid is useful to diagnose an inflammatory state, such as retinitis uveitis, Behcet’s syndrome, and diseases such as glaucoma. The drop in the normal nitrite level (nitrite level in healthy control ≈ 120 μmol L^–1^; in diseased ≈ 89.29 μmol L^–1^) caused particularly *via* oxidation of tear nitric oxide to peroxynitrite in Behcet’s patients can be determined by measuring the concentration of tear nitrites and oxide byproducts.^136^ The nitrite ions react with sulfanilamide to give diazonium salt that in turn binds with *N*-(1-naphthyl)ethylenediamine dihydrochloride yielding pink color azo dye whose absorption intensity falls in the visible region (528 nm).^128^ Since intensity of the pink color is directly proportional to the concentration of nitrite ions, the light pink color can point to infection from uveitis. The sensor fabricated by Moreddu *et al.*(4) possessed a good sensitivity of 0.03 nm/(μmol L^–1^) of nitrites and a LOD of 24.4 μmol L^–1^ (Figure 8G(iv),H(iv)).^4^ The same group also fabricated a contact lens sensor to monitor corneal temperature using thermochromic liquid crystals (TLCs) which exhibit a reversible color change to temperature. The temperature sensor can become a potential replacement for the electric sensor with an accuracy of up to 0.1 °C, quick response time, and multiple color transitions with excellent reversibility.^7^ Chloride ions’ presence can be determined by the color change from colorless to blue color when Hg^+^ from mercuric 2,4,6-tripyridyl-*s*-triazine reacts with Cl^–^ to yield HgCl_2_. Despite the advantages such as quick response time (1–3 min) and short wearing time (30 min), usage of a mercury compound may pose health threats.^120^ Sodium ion (Na^+^) concentration was successfully sensed using contact lens with laser inscribed holographic nanostructures, to measure the extent of syndrome in its primitive stage.^137^

In general, microfluidic sensor setup with the capability to analyze multiple analytes simultaneously proved to be reliable through *in vitro* studies. However, the concerns such as insufficient amount of tear fluids for multiple microchannels due to dry eye syndrome and ambiguity in the RGB values from overlapping channels are to be taken in consideration.

## Intraocular Pressure Sensing

Ocular hypertension or elevated intraocular pressure is a detrimental condition for developing glaucoma which might lead to irreversible blindness if unattended over the long run. In the absence of early diagnosis and treatment, ocular hypertension leads to gradual degradation of retinal ganglion cells and their axons causing optic neuropathies.^1^ The asymptomatic development of glaucoma at a slow pace can lead to heavy damage of vision even before its early manifestation, and therefore a close watch over IOP variation is much stressed. A prompt diagnosis of IOP variation and measures to bring down IOP can have control over the development of glaucoma.^2,3,4^ Even in healthy subjects a circadian rhythm of IOP is prevalent,^5−8^ and undulation in IOP is observed in glaucoma patients.^9,10^ Proper quantification of IOP and getting a correlation will be of immense help to know the disease level and its progress so that efficacious treatment can be sought at the earliest. Nocturnal elevation of IOP^6^ and its variation in the course of the wake–sleep cycle,^11^ diurnal-to-nocturnal transition, and postural dependent fluctuation owing to the hike in episcleral venous pressure and circulation of body fluid^12−14^ urge for the continuous or round the clock monitoring of IOP. To date, the reasons for the elevation and the fluctuation of IOP are debatable but the health risks can be avoided by taking effective strategies to treat glaucoma well in advance. The information provided by the traditional techniques on IOP are insufficient and have the limitation in continuous monitoring as round the clock care is essential in diagnosing both the elevation and variation in IOP to treat glaucoma. Such non-invasive and real-time monitoring of IOP can only be achieved solely with contact lens sensors. In the event of wearing a contact lens there will be five major forces and one minor force that come into play on the basis of the position and the conditions of the eye. The five major forces are atmospheric pressure (*P*_1_), hydrostatic pressure (*P*_0_), force of gravity (*P*), lid force (*F*_lid_), and surface tension force (Fσ), and it is important to consider the impact of these forces in designing a contact lens sensor for point-of-care medication. The force of viscosity (*F*_v_) is the minor force, ascribing negligible role with insignificant values of tear viscosity, and therefore it is out of consideration.^138^

### Pioneering Works in IOP Sensing

Goldmann applanation tonometry (GAT)^139^ is the most widely used measurement method for IOP monitoring, but it demands the patients to be anaesthetized. Earlier IPO was directly measured using ocular implants, and later contact lens based sensors emerged as non-invasive and continuous monitoring gadgets. The heavy metallic structure based recording tonometer was created in 1957 by Maurice^140^ intended to continuously monitor pressure; however, it had limited advantage since it was neither convenient to the patients nor portable in nature. The first tonometer introduced in 1962 is an inspiration from the design of Donder intended to measure IOP on the sclera.^141^ In 1962 Collins^142^ introduced a wireless sensor device by embedding a couple of planar coaxial coils into a soft contact lens serving as a passive resonant circuit which operated using a coupled magnetic field and showed a variation in the resonance depending on the IOP difference, but it required surgical implantation. From then on the relentless quest for bringing a truly cost-effective non-invasive IOP sensor led the researchers to bring several alternative methods including microfabricated strain gauges embedded in a soft contact lens, wireless techniques^143,144^ by integrating capacitors,^43^ amperometric glucose sensor incorporated within contact lenses,^44^ and fully integrated RF-powered sensor supported by an LED display; however, none of them turned out be a completely non-invasive device for IOP monitoring.

### Strain Gauge Based IOP Sensors

Strain gauge based IOP sensors avail ample opportunities for the incorporation of a variety of materials for application and being operated with thermal compensation.^47^ Rectifying the limitations of the above-said pioneering devices, in 1967 Gillman and Greene^145^ brought out the first non-invasive contact lens based device with a strain gauge embedded in it. The change in the curvature of cornea based on IOP variation is converted into electrical signal by strain gauges from which IOP is measured.^47^ The sensor was made up of a passive resonant coil/capacitor combination. The IOP was measured by fixing the device over the meridional angle of the corneoscleral junction. Then, the angular change arising from IOP variation was detected. Despite being a non-invasive device, it did not gain popularity as it was expensive. The lens had to be custom-made for every wearer to detect changes in meridional angle. Cooper and Beale^146^ introduced an external method of IOP monitoring by detecting the deformation in the meridional angle using wired telemetry and compared it with commercial strain gauges.

**Figure 9:**
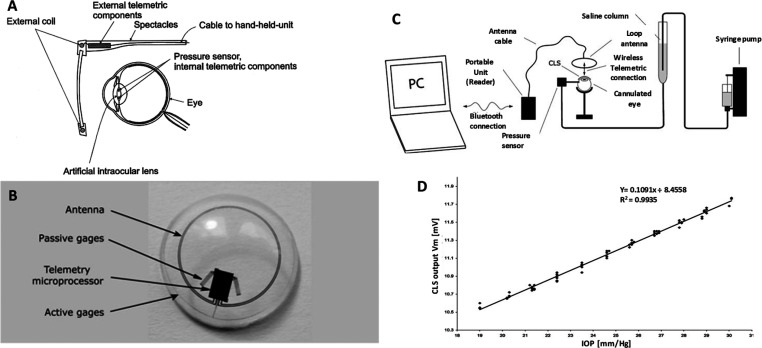
Different tethered IOP sensors, their design, and measurement protocol. (A) Schematic presentation of a system for measuring the intraocular pressure continuously using transponder components. Reproduced with permission from ref (147). Copyright 2000 Elsevier. (B) Silicone soft contact lens sensor showing the location of the sensor-active strain gauges and the sensor-passive strain gauges for thermal compensation for wireless powering and communication, a microprocessor, and an antenna embedded into the soft contact lens; (C) Setup for measuring intraocular pressure wirelessly and (D) plot of intraocular pressure voltages of the output signal of the contact lens sensor (*V*_m_) showing a high linear behavior [linear regression coefficient (*R*^2^) = 0.9935] and a reproducibility of ±0.2 mmHg (95% confidence interval). Reprinted with permission from ref (144). Copyright 2009 John Wiley and Sons.

Taking the advantage of the insensitivity of silicon-based materials toward hydration, Schnakenberg *et al.*(147) reported a silicone-based artificial soft lens sensor system comprised of a pressure sensor connected with transponder components. The readout system was supported by an external transponder incorporated into a spectacle attached to a hand-held unit as seen in Figure 9A. The IOP measurement was carried out both in wired and wireless modes by connecting the pressure sensor to a microwire and to transponder components, respectively, and embedded into a soft contact lens. IOP measurements carried out in both modes exhibited appreciable accuracy on par with the accepted gold standard. The correlation between IOP and corneal curvature was established by Leonardi *et al.*(68) using a soft contact lens housing microfabricated strain gauges, and experimental results reported about 3 μm variation in the radius of the central corneal curvature for an IOP variation of 1 mmHg.^148,149^ Continuous IOP monitoring will benefit the patients in managing glaucoma and in administering drugs to reduce IOP since even standard clinical follow-up reviews are not capable of recognizing peaks and IOP variations continuously.

The successful strain gauge based non-invasive IOP monitoring study in 2004 by Leonardi *et al.*(68) utilized a microfabricated strain gauge embedded soft contact lens^150^ to record changes in corneal curvature/spherical deformations of the eyeball and correlated it to the difference in IOP. The typical sensing device is made by sandwiching a strain gauge comprising a thin microfabricated platinum–titanium (200 nm/(20 nm of Ti)) between two layers of insulating polyimide that serves as flexible carrier material providing protection. The device was fixed on an enucleated porcine’s eye, and to induce controlled variation in IOP, the eye of the animal was cannulated with a butterfly needle positioned in the posterior chamber and connected to a saline bag through a silicone tube and to a syringe pump. For comparison, a pressure sensor is inserted in the silicone tube and the resulting output is compared with the signals from the contact lens sensor. Though this setup was non-invasive, it was still not wireless, and the transformation from wired to wireless remained technically challenging and complex. They imitated a very similar sensor setup and made it wireless by housing a microprocessor and an antenna in the lens and used an application-specific integrated circuit (ASIC) for wireless communication, making it a passive telemetry system for continuous monitoring of IOP.^144^ The digital image of the contact lens sensor and the experimental setup are shown in Figure 9B,C, respectively. The *in vivo* studies were carried out on enucleated pig eyes under simplified physiological conditions. The plot between the output signal of the contact lens (*V*_m_) *vs* IOP showed a high linear behavior with a linear regression coefficient of (*R*^2^) = 0.9935 and a reproducibility of ±0.2 mmHg (Figure 9D. This wireless method could monitor IOP up to 24 h regardless of the patient’s position, activities, and involvement. A Wheatstone bridge circuit using a metal electrode was used as a strain gauge sensor to measure IOP non-invasively with a high sensitivity of 20 μV/mmHg.^151^ Passive strain sensors comprising a variable inductor and a constant capacitor with *ex vivo* trials on a canine eye come more economically and simply in design.^152^ Other non-invasive sensing platforms with islets transplantation further facilitate IOP monitoring.^153^ These methods are versatile in terms of using a list of materials surpassing the interference of temperature-based effects.

### Graphene-Incorporated Strain Gauge IOP Sensors

**Figure 10:**
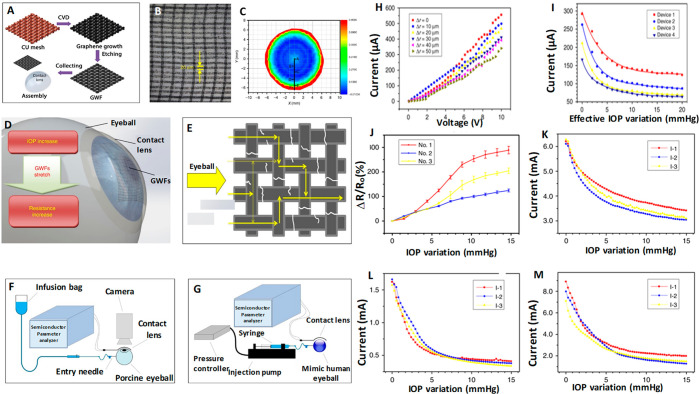
Graphene-incorporated strain gauge IOP sensors, fabrication process, images, sensing mechanism, and results. (A) Schematic for process of graphene woven fabric (GWF) based IOP device fabrication. (B) Digital image of the GWFs. (C) Strains variation with the intraocular pressure. (D) Working principle of the device. (E) Current pathway through a fractured graphene woven fabric (GWF). (F) Setup for the mechanical testing and *in vitro* application experiments. (G) Schematic for the sensitive performance testing. (H) Current–voltage relationship of the device. (I) Relationship between the current and IOP increasing under 10 V of the four devices. (J) Relationship between the resistance change rate and the IOP variation. (K–M) Relationship between the IOP variation and the current when keeping the voltage constant in 10 V. Reprinted with permission from ref (1). Copyright 2019 Springer Nature.

Utilizing the sensing ability of graphene oxide, graphene woven fabric (GWF) had been used as a standalone intraocular pressure sensing material in contact lenses without additional nanostructured components^1^ as it possesses significant sensitivity toward strain, strechability, and flexibility.^154,155^ Moreover GWF’s biocompatibility and transparency (>80%) satisfy the conditions to function as a strain sensor for a tonometer which assures delivering cost-effective contact lens sensor. The GWF was fabricated on copper (Cu) mesh substrate and template by chemical vapor deposition, and the obtained product along with Cu mesh was immersed into a mixture of FeCl_3_/HCl (1:1 (mol L^–1^)) for a period of 2 h to remove Cu mesh and get GWF (Figure 10A–C. Due to the homogeneous hydrophilicity GWF closely adheres to the cornea, thus making GWF a potential sensing material for intraocular pressure monitoring. An increase in IOP causes a small deformation of the eyeball and eventual elongation of the contact lens and the GWF attached on it, as shown in Figure 10D,E, respectively. On the basis of the IOP increase and the stretching of GWF, the system exhibited a hike in resistance and *vice versa*. The current change observed under the constant voltage due to variation in IOP helped to monitor IOP. IOPs were calculated from the correlation between the change in resistance and the deformation and the relationship between current and voltage. The sensitivity for the device fabricated was on a model of a human eyeball fitted with a syringe pump and tested, and *in vitro* experiments were conducted on porcine eyes (Figure 10F,G. The rate of change of the resistance under different IOPs, the relationship between the change in current, and variation in IOP for constant voltage were recorded using four devices (Figure 10H–M). The test results proved that GWF showed high sensitivity toward the IOP induced deformation of the eyeball which causes strain of the contact lens. Xu *et al.*(156) used few-layer graphene to construct a biocompatible strain gauge sensor with an accuracy to sense up to 150 μV mmHg^–1^ to serve around the clock IOP monitoring. Therefore, a few layers of graphene and GWF are highly sensitive to strain sensing, a promising potential cost-effective contact lens with lower power compared to other devices.

### Transducer- and Microinductor-Based IOP Sensors

Corneal curvature changes due to the difference in IOP in turn cause inductance changes in transducer and microinductors in the contact used as sensing material, and IOP is calculated from the change in the inductance. Couvillon *et al.*(157) used the principle of applanation tonometry and designed a device by embedding a circular applanate along with a pressure transducer in the hydrogel-based contact lens and monitored IOP without obstructing the vision. McLaren *et al.*(158) designed a battery-powered wired device embedded with a commercial telemetric pressure sensor transducer and implanted it subcutaneously on the dorsal neck between the scapulae of pigmented rabbits. A fluid-filled catheter was implanted in the anterior chamber *via* a limbal opening which conducts pressure to the transducer. The pressure was recorded by the transducer, and the information broadcast by amplitude radio is received by the receiver antenna. Seven rabbits were subjected to IOP monitoring for a period of 180–370 days for 15 s every 2.5 min. The main advantages of this kind of telemetry-based IOP are the viability of measuring IOP in the absence of the investigator and under open and closed eyelids conditions. Puers introduced PMMA-based contact lenses incorporating an implantable hybrid integrated transponder with a bulk micromachined pressure sensor.^159,160^ Taking advantage of silicon micromachining of miniaturizing transducers into the sub-millimeter range, long-term implantation of small microdevices can be envisaged by coupling low-power integrated circuits.^147^ Microinductor measurement based wireless IOP sensors was developed using MEMS fabrication technology. The sensor was comprised of a sensing inductor coil, radiofrequency integrated circuit (RFIC), and antenna metal film structure. The microinductor setup was coated with parylene-*C* and embedded into a soft contact lens made of hydroxyethyl methacrylate (HEMA) by cast-molding method. There was good agreement between the measurement results of the microinductor inductance and simulation results for microinductor radius variation and oscillation frequency.^2^

### Capacitive Sensors

**Figure 11:**
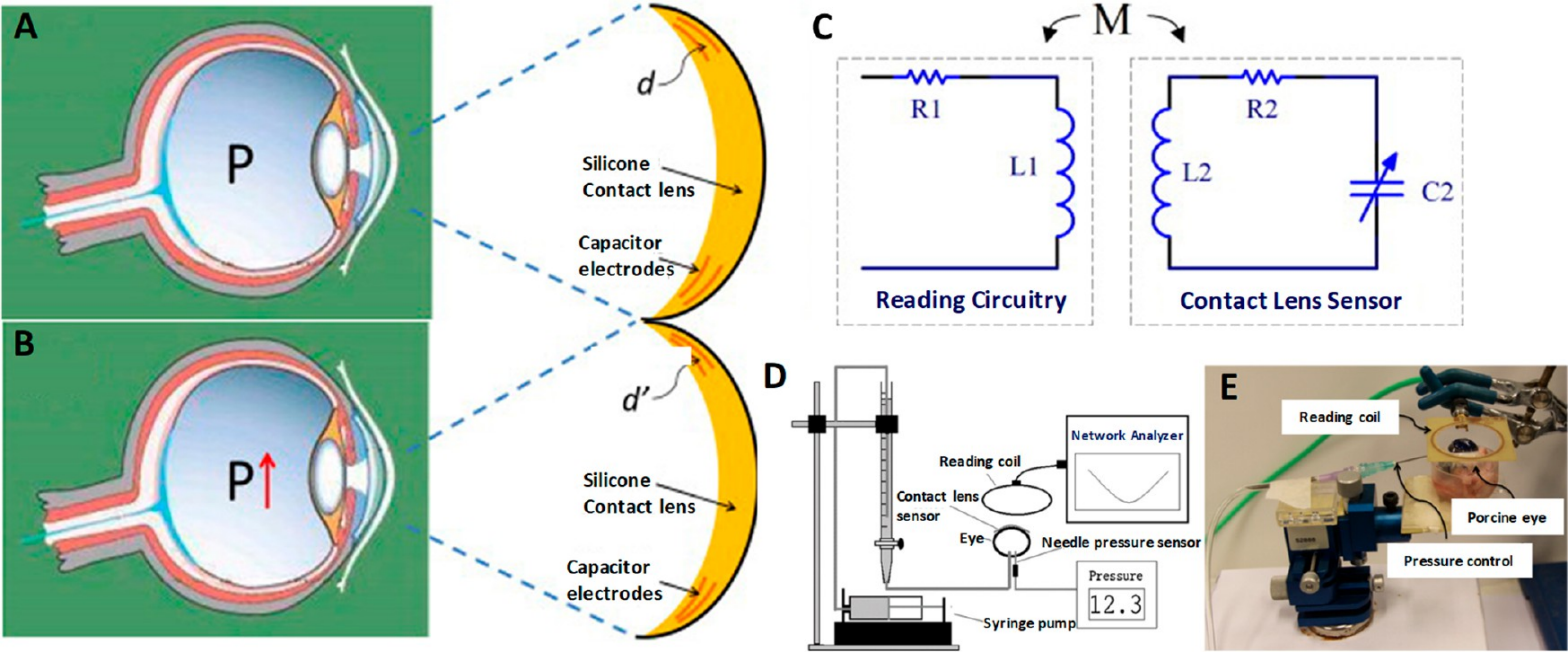
Capacitance-based IOP sensor, design, working principle, and results. (A) Capacitance-based contact lens sensor configuration for IOP measurement. (B) Contact lens sensor configuration on eye with high IOP (IOP fluctuations change of corneal curvature exhibited by the change in the capacitance indicated by the change in the distance between the electrodes). (C) Schematic illustration of reading circuitry of the contact lens sensor. (D) Scheme of sensor testing setup. (E) Photograph of sensor testing setup on porcine eye. Reprinted with permission from ref (43). Copyright 2013 Elsevier.

These devices consist of a sensing (inner) and a reference (outer) layer, each comprised of an inductor–capacitor unit. Any change in the curvature of the cornea induces a change in the resonance frequency of the inductor–capacitor circuit. The induced change in the resonance is detected by the sensing layer with respect to the reference layer and associated with the variation in IOP. This technique is befitting for low-force applications only.^47^ Backlund *et al.*(161,162) adopted the concept of collings and proposed constructing a passive telemetry unit by placing a bulk micromachined capacitive pressure sensor parallel to a wound inductor coil and housing this unit into an artificial intraocular lens. The wireless interrogation of the resonance frequency was assisted by a small antenna fixed with a spectacle frame. The results obtained from the *in vitro* experiments are in accordance with theoretical predictions. Though piezoresistive strain gauge sensors and capacitive pressure sensors are mostly used for the IOP monitoring,^163^ capacitive pressure sensors are more apt for low-force application because they are highly sensitive to pressure change and consume low power.^164,165^ They are mostly used separately, but an attempt was made by Chen *et al.*(43) coupled a capacitor with an inductive coil to form an inductor–capacitor (LC) resonance circuit to be curvature sensitive and housing it inside a silicon contact lens. The resonance frequency was
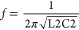
5where L2 is the inductance and C2 is the capacitance of the resonance circuit. The lens consisted of both hard and soft silicone lenses. The hard silicone lens is the upper part of the device embedded with the reference layer comprising the inductive coil and the upper capacitor electrode, while the soft silicone lens had the sensing layer made of the lower electrode (Figure 11A–E). The IOP measurement is carried out on the basis of the mechanical relationship between the curvature of the lens and IOP. When IOP increases, it induces an expansion of the corneal curvature which causes an increase in capacitive gap spacing due to a change in the capacitance observed by the sensing layer reflecting in the resonance frequency and the reverse happens when IOP lowers. The tests were conducted both on the silicone rubber model and porcine eyes for IOP monitoring. The sensor was non-invasive and capable of continuously monitoring IOP with a high sensitivity (>200 ppm/mmHg in porcine eyes tests) with good linearity (*R* > 0.997 in porcine eyes tests). This method is simple and advantageous since it has wireless readouts and passive, but the challenges involved with *in vivo* testing on animals are not addressed.

### Multifunctional Sensors

**Figure 12:**
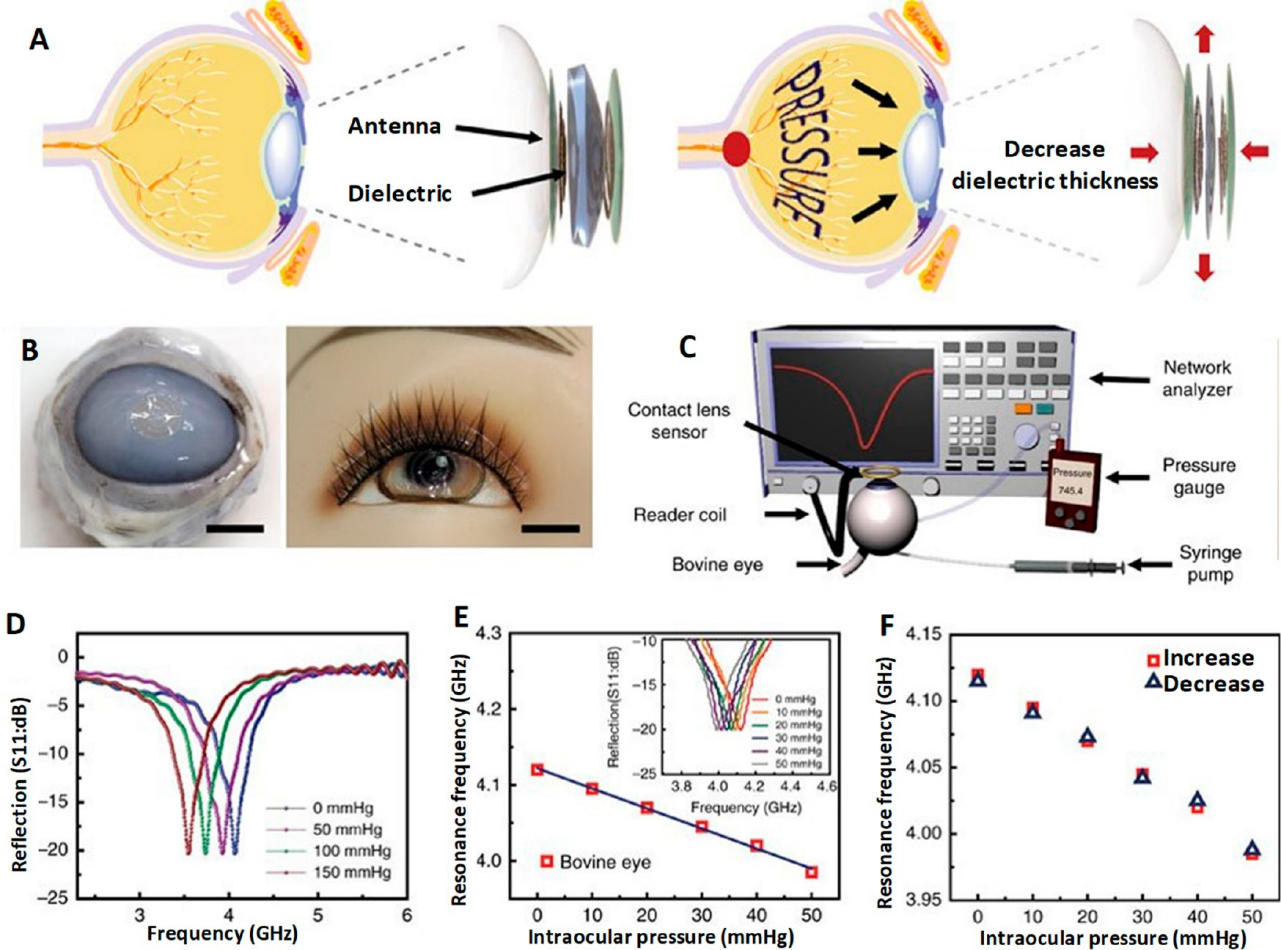
Graphene–AgNW hybrid electrodes incorporated IOP sensor, fabrication, working principle, and results. (A) Schematic showing the mechanism AgNWs spiral coil based intraocular pressure sensor. (B) Photographs of the sensor transferred onto the contact lens worn by a bovine eyeball (left) and a mannequin eye (right). Scale bar, 1 cm. (C) Schematic of the experimental setup for wireless intraocular pressure sensing. (D) Wireless recording of the reflection coefficients at different pressures. (E) Frequency response of the intraocular pressure sensor on the bovine eye from 5 to 50 mmHg. (Inset: corresponding reflection coefficients of the sensor). (F) Frequency response of the sensor during a pressure cycle. Reprinted with permission from ref (40). Copyright 2017 Springer Nature.

Most contact lens based sensors reported are capable of monitoring either glucose or IOP but not both. In the case of a patient with more than one disorder, single analyte sensing contact lenses do not meet the requirements. Such circumstances demand a multitasking contact lens having the advantage of performing multisensing. The multifunctional sensor based contact lens developed by Kim *et al.*(40) circumvents the limitation found in the other single analyte sensing prototype devices. This sensor is able to continuously monitor intraocular pressure along with glucose in tear fluid simultaneously, yet provide independent results. The electrical signals generated as a response of the mechanical strain furnishes information for the reliable operation of the devices.^166^ The device for monitoring the intraocular pressure was fabricated by coating of the copper (Cu) foil with the parylene, followed by spin coating and annealing AgNW on the parylene substrate. Etch-back and ion etching process were used for patterning the AgNW spiral coil. Thus, both bottom and top AgNW spiral coils were formed following the above process and spin coated with ecoflex (silicone elastomer) and an additional layer of parylene that serves as the passivation layer. Nickel (Ni) etchant was used to etch the bottom copper foil. The device was incorporated with the contact lens, and the permeation of oxygen and water was facilitated by punching the central area of the sensor. The sensor was made and incorporated into the contact lens as described above where a sandwiched structure is made of a couple of ecoflexes between the two graphene-AgNW hybrid electrodes wound into a spiral shape, as shown in Figure 12A. In this work, only *in vitro* wireless monitoring of intraocular pressure was carried out on bovine eyeballs and the sensor exhibited transparency adequately on the bovine or mannequin eye, ensuring clear vision (Figure 12B. The corneal radius of curvature undergoes an increase at high values of intraocular pressure (*P*) which increases the inductance and capacitance shifting the spiral antenna′s reflection spectra to a lower frequency (*f*),^167^ indicating *f*_sensor_α × 1/√*P*. At lower pressure (>50 mmHg) the frequency response is linear and this linearity decreases at high pressure. The above-described senor system demonstrated reproducible results with consistency. Therefore, this wearable device might come in handy for sensing two analytes simultaneously (Figure 12C–F).

### Microfluidics-Based IOP Sensors

**Figure 13:**
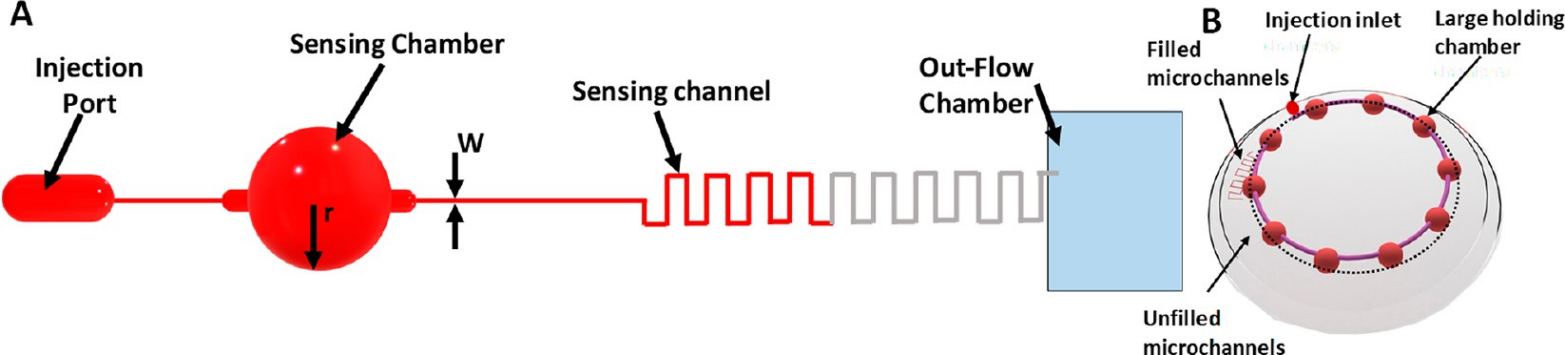
Basic principle and components of microfluidic IOP sensor and fabrication of contact lens with IOP sensor. (A) Schematic illustration of calibration device. (B) Digital image of the microfabricated PDMS based device.

Microfluidic contact lenses do not involve opaque electronic components, buckled deformations, and surface mounted rigid materials. This makes microfluidic contact lenses be free of warpage and harming cornea or eyelid. The possibility of housing more than one sensing module to detect multiple analytes and the accuracy in sensing makes it a more preferred choice over soft contact lenses with electronic components incorporated.^120^ The detection of analytes such as glucose, urea, and chloride ion is made out from a distinguishable color change manifested by the sensing module. IOP can be detected from the displaced dyed liquid resulting from the deformation of the sensing chamber. Silicone hydrogel materials with a thermoplastic nature are useful in fabricating microfluidic contact lenses. Of these, PDMS is one of the most commonly used silicone hydrogel materials because it has good physical and chemical properties and it is biocompatible, too. Yan^168^ adopted Laplace’s principle and soft lithographed a glycerol-based microfluidic contact lens wireless and non-invasive sensor with PDMS for pressure measurement. The device possesses (Figure 13A,B) a large sized sensing chamber network of height *H* and radius *r* in connection with a sensing channel of width *w* and height *h* and an out-flow chamber of very high volume to function as a pressure relief conduit. A bottom-up approach of micromachining, replica-molding, and oxygen plasma is adopted to embed the senor into the polymer network, and the color fluid is injected *via* fluid injection. The sensing channel serves as a sensing element, and when the sensing chamber experiences elevated pressure, the elastomer unit receives the induced strain and an internal pressure created by the entire microfluidic network. This causes a compression of the sensing chamber and fluid of certain volume flows into the incompressible microchannel. By observing the displacement or the wetted length of the colored liquid using a digital microscope, the pressure can be calculated. In the event of a negative pressure the colored liquid travels back to the sensing chamber from the sensing channel. This withdrawal of the liquid is attributed to the recovery properties of the polymer.

Araci *et al.*(9) constructed a passive microfluidic pressure sensor by integrating an airtight microfluidic channel with two ends. One end is linked to a gas reservoir, and the other end of the channel has the approach to the aqueous intraocular liquid. A combination of capillary force and intraocular pressure displaces the liquid into the microchannel until it attains the equilibrium point. A contact lens sensor was fabricated by incorporating the above-described sensing unit and can be used either as an independent device or implanted in the eye in the course of a surgery. The IOP increase shifts the liquid–water interface to the gas reservoir end, and the displacement is photographed and analyzed using software to get the measure of pressure. This passive device with proven reproducibility and high precision favors the wearer by providing easy self-examination with mere visual readouts.^118^

**Figure 14:**
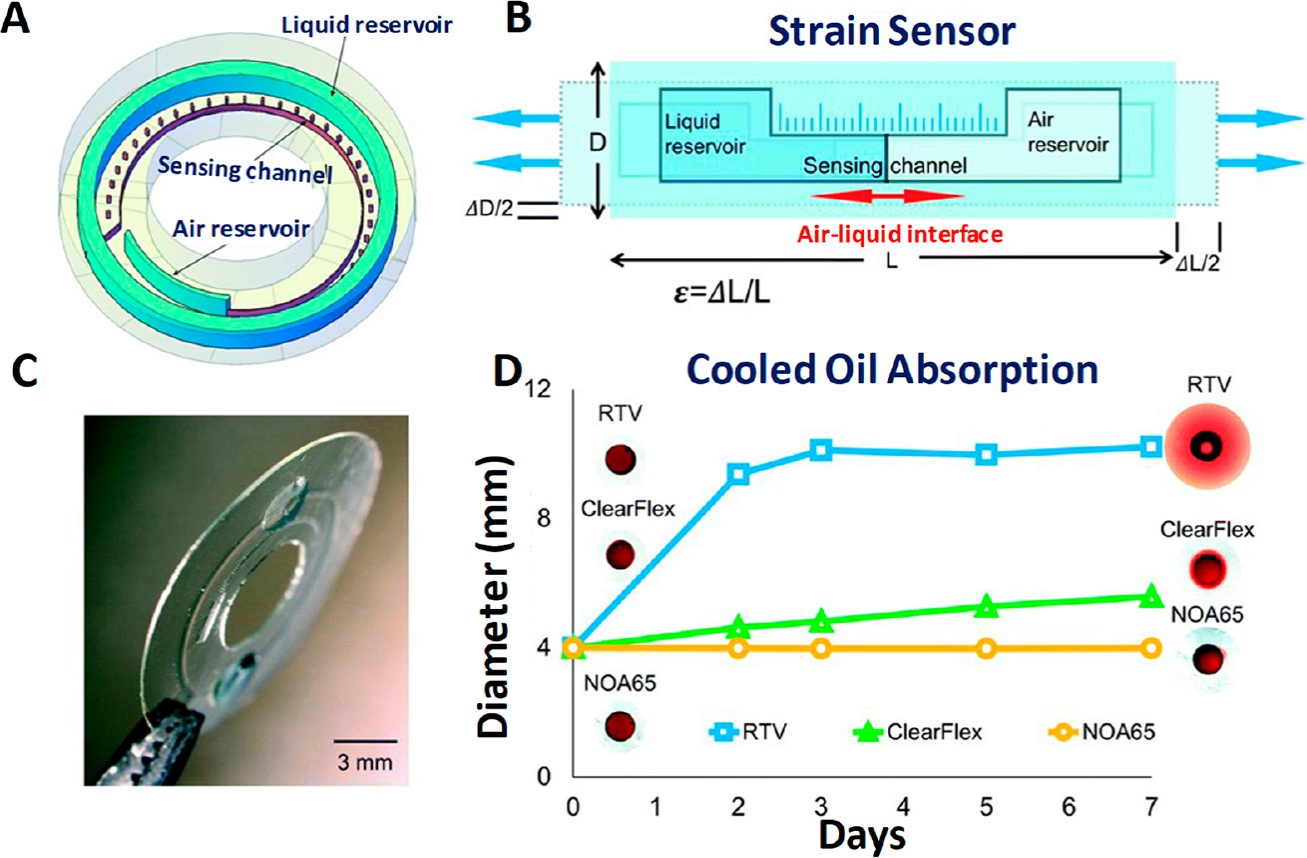
Microfluidic pressure sensor working principle, incorporation in contact lens and results. (A) 3D schematics of the strain sensor. The sensor is composed of a liquid reservoir, an air reservoir, and a sensing channel. (B) Cartoon sketch showing the strain sensor operation principle. (C) Photograph of the wearable microfluidic strain sensor (150 μm thickness). (D) Results of the dyed oil absorption experiment for different materials, namely, RTV (PDMS), Clearflex, and NOA65. The inset shows the microscope images of the wells fabricated from corresponding materials comparing the initial and the final states of dyed oil absorption. Adapted with permission from ref (169). Copyright 2018 The Royal Society of Chemistry.

Measuring the volume expansion and making a correlation can give the pressure readout, because physical displacement of the air–liquid interface per 1 mmH change is the measure of sensitivity. Therefore, in the microfluidic pressure sensor the sensitivity is proportional to the ratio of the reservoir volume to channel cross-section. Thus, the width of the channel should be a minimum to construct a highly sensitive sensor to IOP monitoring.^13,169^ The general principle of any microfluidic strain sensor depends on detecting inductance or capacitance and resistance caused by mechanical deformation,^170^ and Agaoglu *et al.*(169) fabricated a passive integrated microfluidic sensor based on a transduction mechanism that accounts for the large volume change of the fluid, in response to a small change in strain. The reported sensor has a sensing channel in the middle and is connected to liquid and air reservoirs on either end (Figure 14A–C). The displacement of the air–liquid interface as an effect of vacuum due to either the axial stretching or releasing corresponds to the pressure. The polymer material making up the lens should be compatible with guide liquid to be free from hysteresis^13^ and based on weeklong dyed oil absorption experiment clearflex and NOA65 became the material of interest with their enhanced oleophobicity but PDMS is ignored due to its oleophilic nature (Figure 14D).^171,172^ With the high detection range of <0.06% for uniaxial and <0.004% for biaxial strains and the smartphone operated passive nature, this technique suits easy self-monitoring of IOP by the wearers. Moreover the device was quite reliable, as it performed more than 19 h of continuous operation and exhibited a prolonged life (beyond 7 months) and there was an acceptable agreement between simulation and experimental results.^169^ The dyed oil absorption experiment can provide guidelines for the choice of compatible materials.

**Figure 15:**
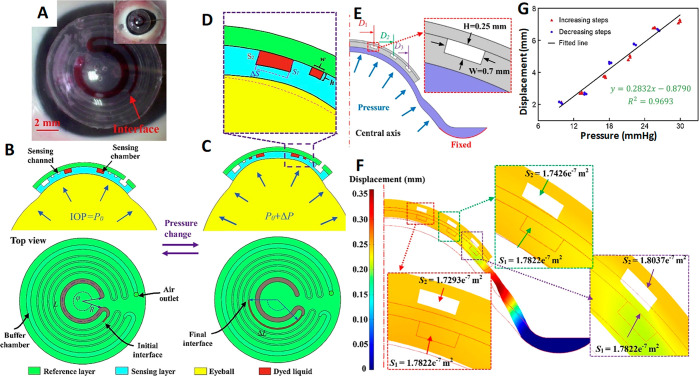
PDMS- and PET-based microfluidic contact lens for IOP measurement: (A) Digital images of fabricated microfluidic contact lenses using PDMS and PET, worn on the porcine eye *ex vivo*. (B, C) Sectional view and top view of microfluidic contact lenses under different IOPs of *P*_0_ and *P*_0_ + Δ*P*. (D) Sectional area change of sensing chamber. FEM results of microfluidic contact lens: (E) Simulation illustration. (F) Sectional view of the sensor with a pressure of 40 mmHg in relation to deformation. (G) Variation of displacement and IOP during three cycles of increasing and decreasing pressure. Reprinted with permission from ref (48). Copyright 2019 Elsevier.

A five step (soft lithography, silanization of the substrate, plasma-treated bonding, thermoforming, and liquid injection) assisted microfluidic contact lens IOP sensor is fabricated using a combination of PDMS and PET (Figure 15A–F).^48,49^ Micropatterned PDMS served as sensing layer and a hard PET reference layer constituted the sensing chamber, sensing channel, and the buffer chamber. The red colored liquid filled one-half of the sensing channel, and the other half was filled by the dyed glycerol and the sensing chamber is filled with a colored liquid. The sensing chamber undergoes a reduction of its volume with the IOP increase, and that in turn displaces the dyed liquid’s interface in the sensing channel and the reverse occurs with a decrease of IOP. The displacement of liquid interface (Δ*l*) and sensitivity (*t*) can be calculated with the change in displacement, using a smartphone camera. The influence of various parameters on the sensing mechanism is studied using the finite element modeling (FEM). A linear correlation between the liquid displacement and IOP was presented, with a high sensitivity of 0.2832 mmHg^–1^ in a range of 832 mmHg was obtained through *ex vivo* clinical tests, performed on porcine eyes with good reproducibility, reversibility, and prolonged stability (Figure 15G).

The decline in the sensor’s sensitivity with wide microchannels and the signal-to-noise ratio are serious concerns to be rectified in microfluidic sensors. High signal-to-noise ratio is usually found in black silicon^173^ or gold nanodot^6^ based optical cavity pressure sensors. Microfluidic sensors with multiple liquid reservoirs in concentric fashion offer enhanced signal-to-noise ratio by overcoming the rapid fluctuations from IOP induced strain.^174^ More recently microfluidic sensors using photonic crystals as colorimetric sensing units with microhydraulic amplification mechanism provide non-invasive smartphone assisted IOP monitoring, eliminating the role of optical spectrometer with LODs of 3.2 and 5.12 mmHg.^175^ Microfluidic contact lens based IOP sensors eliminated electronic components and have proved to be a completely reliable passive tool for the close monitoring of glaucoma. Further investigation to draw a relationship between the internal pressures of an object with its acoustic refection coefficient can take a lead in IOP management using a smartphone laser.^176^

## Contact Lens Based Drug Delivery Systems

Eye drops are a non-invasive and nonsurgical treatment option for ocular treatment. The eye is an elaborate and complex organ possessing different anatomical and physiological barriers such as the precorneal and corneal barriers, the conjunctival barriers, the blood–aqueous barrier, and the blood–retinal barrier,^177,178^ all of which limit drug penetration. Therefore, effective drug delivery for ocular infections and diseases remains quite challenging since a major portion of the instilled eye drops are rapidly swept out of the ocular surface due to precorneal eliminations including tear turnover and drainage causing both underdosing with insufficient quantity of the drug and unpleasant side effects.^179^ In order to enhance prolonged drug contact with the cornea, administration of ophthalmic dosage in the form of viscous solutions and ointments, gels, and usage of contact lens have been suggested for anterior segment diseases.^180,181^ Furthermore, clinical studies conducted showed that the contact lenses immersed into antibiotics possessed higher drug penetration ability than subconjunctival injections,^182^ making contact lenses an efficient therapeutic gadget. Contact lenses can enhance ocular drug delivery with prolonged residence time of the drug in the cornea/target tissues and sustained release of the drug with appropriate control over timing. The localization on the cornea^183−187^ and blending the advantages can greatly improve the usage of therapeutic contact lenses. However, the poor drug loading capacity of conventional contact lenses curtail them from being employed for therapeutic applications, causing serious challenges for the anchorage of the drugs on to the lenses.^188,189^

While drug-soaked contact lenses can exhibit a burst drug release property, unique contact lenses developed using molecular imprinting or having microparticles embedded in them showed sustained drug release and tailoring these features can ensure improve cost-effective drug delivery by means of low drug dosage requirement, avoiding frequent administration of the drug and reducing the drainage loss.^183^ The contact lens used for therapeutic purpose should absorb sufficient load of drug when soaked in drug solution and release the absorbed drug in a controlled manner.^191^ The initial reports of drug-eluting contact lenses with drug loading by soaking lacked sustained release by releasing all the absorbed drugs within 1–3 h.^192−194^ To remedy this drawback, the contact lenses used for drug release are developed using molecular imprinting technique by incorporating drug-loaded implants and colloidal micro- and nanoparticles and sustained drug release can be made possible by involving, supercritical fluid technology,^195^ ionic interactions, and vitamin E diffusion barriers.^195−201^ Molecular imprinting method can drastically increase the drug loading capacity of the contact lenses in several-fold and the matrix composition of the contact lenses does reflect in the adsorption affinity of the drugs.^191,202,203^ For the enhancement of sustained drug release, key factors are the resistance to mass transport at the interface between the matrix of the hydrogel lens and drug-loaded vehicles and Fick′s law of diffusion.^200^

**Figure 16:**
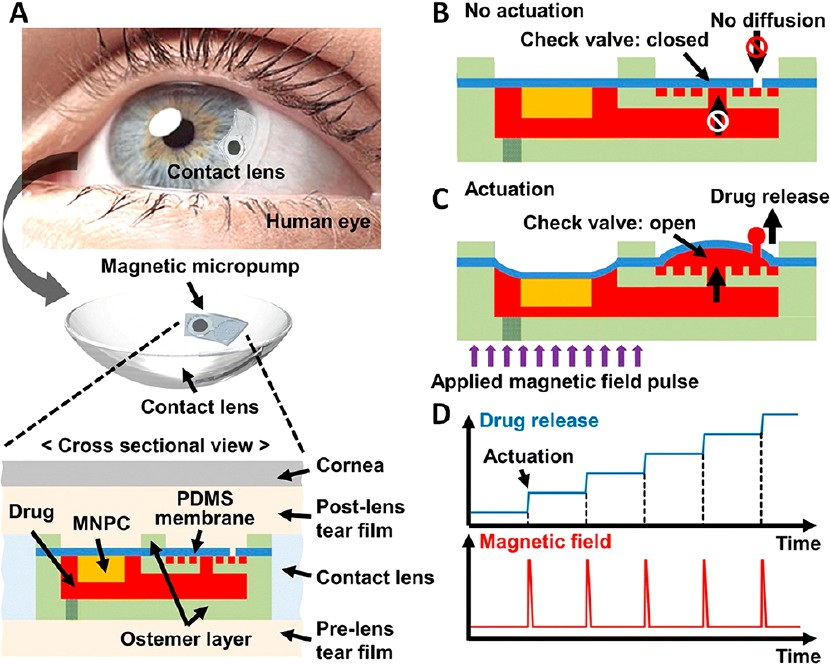
(A) Schematic representation of the proposed thin magnetic micropump integrated in contact lens. (The cross-sectional view shows how the drug can be released from the micropump into the postlens tear film through the aperture in the PDMS membrane). (B, C) Working principle of the proposed micropump: status of the micropump under no actuation and actuation of magnetic field pulse, respectively. (D) Resulting on-demand drug release under an external controllable magnetic field pulse. Reprinted with permission from ref (190). Copyright 2020 Springer Nature.

Nanoscape polymeric nanoparticles are preferred over the microscale since larger sized particles can have parasitic effects on optical and physical properties.^204^ The drug-eluting contact lenses are manufactured by synthesizing the drug-loaded polymeric nanoparticles first, followed by embedding them into the polymer matrix^197^ by mixing them along with the monomers prior to polymerization. If the drugs to be incorporated are susceptible to degradation when exposed to UV or high temperatures, then the drug loading can be made successful by soaking the polymerized contact lens into solution containing the drug molecules.^204^ Wang and Park^190^ explored a contact lens with an on-demand drug delivery system, where a thin magnetic micropump is incorporated in the contact lens. The micropump with magnetic nanoparticle–PDMS composite (MNPC) for actuation has a check valve which is operated using an external magnetic field and not battery powered. On-demand drug supply can be met by applying an external magnetic field to open the check valve and withdrawing the magnetic field closes the valve, and the drug dosage is controlled by altering the frequency and the strength of the magnetic field (Figure 16A–D).

Thus, the method to load the drugs is chosen on the basis of the nature of the drug. Different modes and adaptations to load and release some important drugs are discussed below.

### Contact Lenses for Timolol Delivery

Timolol/Timol is a β-blocker drug used for treating IOP and glaucoma, in addition to high blood pressure. The contact lenses to be used for treating glaucoma should be recyclable and have the ability to have sustained release of Timololin in a controlled fashion during the day and refill during the night, by being soak in Timolol.^191^ Weakly cross-linked hydrogels such as hydroxyethyl methacrylate (HEMA) hydrogels with high water content enhance diffusion of solutes and oxygen; however, the contact lenses made of HEMA have very inadequate uptake of drugs, demanding copolymerization with other monomers to help in molecular imprinting. When synthesizing polymers using molecular imprinting along with the drug to be uploaded, upon removing the template molecules, the polymer is enriched with a large number of cavities, possessing affinity for the drug molecules to be absorbed.^205,206^ Alvarez-Lorenzo *et al.*(191) copolymerized HEMA with hydrophobic methyl methacrylate (MMA) and hydrophilic methacrylic acid (MAA) taken in small quantity. Hydrophilic MAA as copolymer along with HEMA sufficiently enhanced Timolol loading capacity to the required level of therapeutic application. Incorporation of MAA greatly improved the drug loading capacity and the HEMA/MAA combination with 100 mM MAA could absorb 12 mg of Timolol/(g of dry hydrogels). The hydrogels showed better swelling behavior at 37 °C than at 25 °C and Timolol release at 37 °C using 0.095% 03105g dry samples in 12 mL of 0.095 NaCl (pH 5.5), for a period of 48 h, showed the robust nature of the molecular imprinted hydrogels for ocular drug delivery. UV assisted polymerization of *N*,*N*-diethylacrylamide using ethylene glycol dimethacrylate (EGDMA) as a cross-linker along with MAA as functional polymer made imprinted hydrogel exhibit better Timolol loading capacity than the non-imprinted counterpart. There was an appreciable increase in Timolol anchorage above 60 mM EGDMA and a superior behavior with 80 mM EGDMA. A prolonged Timolol release was reported for a period of more than 24 h at 0.9% NaCl aqueous solution.^179,202^ Timolol imprinted lenses have been developed with UV irradiation employing polymers such as *N*,*N*-diethylacrylamide (DEAA), 2-hydroxyethyl methacrylate (HEMA), 1-[tris(trimethylsiloxy)silyl]propyl methacrylate (SiMA), and *N*,*N*-dimethylacrylamide (DMAA) (50:50 (v/v)), or methyl methacrylate (MMA) and DMAA (50:50 (v/v)) solutions using ethylene glycol dimethacrylate (EGDMA, 140 mm) as cross-linker and methacrylic acid (MAA, 100 mm) as functional polymer along with Timolol maleate (25 mm). By fitting the Langmuir equation to the adsorption isotherms obtained at 37 °C in water, the order of Timolol affinity by the hydrogels prepared follows the order EMA > SiMA–DMAA > MMA–DMAA > DEAA.
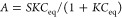
6where *A* is the quantity of Timolol adsorbed per unit volume of the gel, *S* is the maximum adsorption capacity, *K* is the affinity of one adsorption site, *SK* is the overall affinity, and *C*_eq_ is the residual Timolol concentration in the solution at equilibrium. The above equation can be rearranged in order to enhance fitting of experimental data for parameter evaluation.
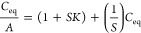
7*S* and *K* can be calculated from the slope and intercept of a plot of *C*_eq_/*AvsC*_eq_.^202,203^ The value of the diffusion coefficient of the lenses proved that Timolol molecules get detached from the hydrophilic networks (MMA–DMAA and SiMA–DMAA lenses) due to its poor affinity. This signifies the importance of choosing the right polymer backbone and their composition to fabricate the lenses with the desired drug loading and drug release profile for the ocular therapy.^203^ To facilitate contact lens enhanced ocular therapy further, prolonged^207^ and temperature-sensitive^208^ drug release lenses were designed where the external stimuli such as pH and temperature play a major role. Timolol, being water-soluble, reacts with propoxylated glyceryl triacrylate (PGT) forming ester bonds,^208,209^ and this phenomenon helped in fabricating the Timolol-loaded PGT nanoparticles embedded contact lenses. A mixture of PGT and Timolol malate were subjected to thermal polymerization, and the Timolol-loaded PGTs were mixed with a precursor solution along with α,ω-bis(methacryloxypropyl) poly(dimethylsiloxane) followed by polymerization carried out in a mold, to get drug-loaded nanoparticles incorporated silicon hydrogels. The contact lenses fabricated from this demonstrated a sustained Timolol release at a constant rate lasting for a month owing to the ester linkage between the PGT matrix and the Timolol. These contact lenses retained their water content and transparency post-inclusion of the Timolol-loaded PGT nanoparticles, and this model was tested on beagle dogs by using commercially available Acuvue Oasys lenses after immersing them in a solution containing drug-loaded nanoparticles.^210^

### Contact Lens Based Treatment for Fungal Keratitis

Keratitis is an infection or inflammation of the cornea that can be subdivided into superficial keratitis and deep keratitis, causing ocular morbidity and blindness. The cornea can be infected by different fungi such as *Fusarium*, *Aspergillus*, or *Candida*. Fungal keratitis is a severe ocular disease. Superficial keratitis refers to infection in the outer layer of the cornea, which gets healed without leaving behind any scar, but deep keratitis is related to the infection of the deep layers of the cornea and upon healing it might cause a scar that may damage the vision on the basis of the location of the scar. The other causes of infection include amoebic keratitis, bacterial keratitis, herpes keratitis, and photokeratitis. Fungal keratitis can lead to severe health issues including morbidity and blindness which requires immediate and effective treatment, and this infection is often reported in developing countries.^37,211^ The therapy for this kind of infection necessitates sustained release of the drug and the drug administered should be acceptable and sensitive without causing any side effects^212^ and treatment remains both challenging and risky.^213^ Though therapeutic keratoplasty (TKP) combined with medical therapy is a common treatment method to treat this infection, it might harden the vision.^214^

The triazole voriconazole (Vor) has been preferred over the existing traditional antifungal agents to treat fungal infections, either through intravenous and oral administration in the form of tablet or liquid suspension. Oral administration is less preferred owing to prolonged testament time, a fortnight to treat esophageal candidiasis^215^ and several months for Aspergillosis^216^ and the side effects associated with them such as fever, vomiting, rash, peripheral edema, diarrhea, respiratory disorder, and visual disturbances.^217^ Though subconjunctival and intrastromal/intraocular injection are thought to be better alternatives with better reach of the drug to the disease site and minimum side effects,^218^ the risks of visual disturbances, harming the liver, and second endophthalmitis^219^ limit them from usage, leaving eye drops as the only option. For treating ocular diseases, eye drops are considered to be an easy, cost-effective, and non-invasive treatment method, in spite of certain demerits associated with them, such as the physiological barriers of the eyes, the eye tissue tolerance to medicines, and poor bioavailability.^220^ The movement of the eye and scouring the nasolacrimal system curtail the reach of the eye drops to the focus areas and demand frequent administration.^221,222^ Hence, to make sure there is effective administration of the drug against such barriers, a suitable delivery platform is crucially important.

**Figure 17:**
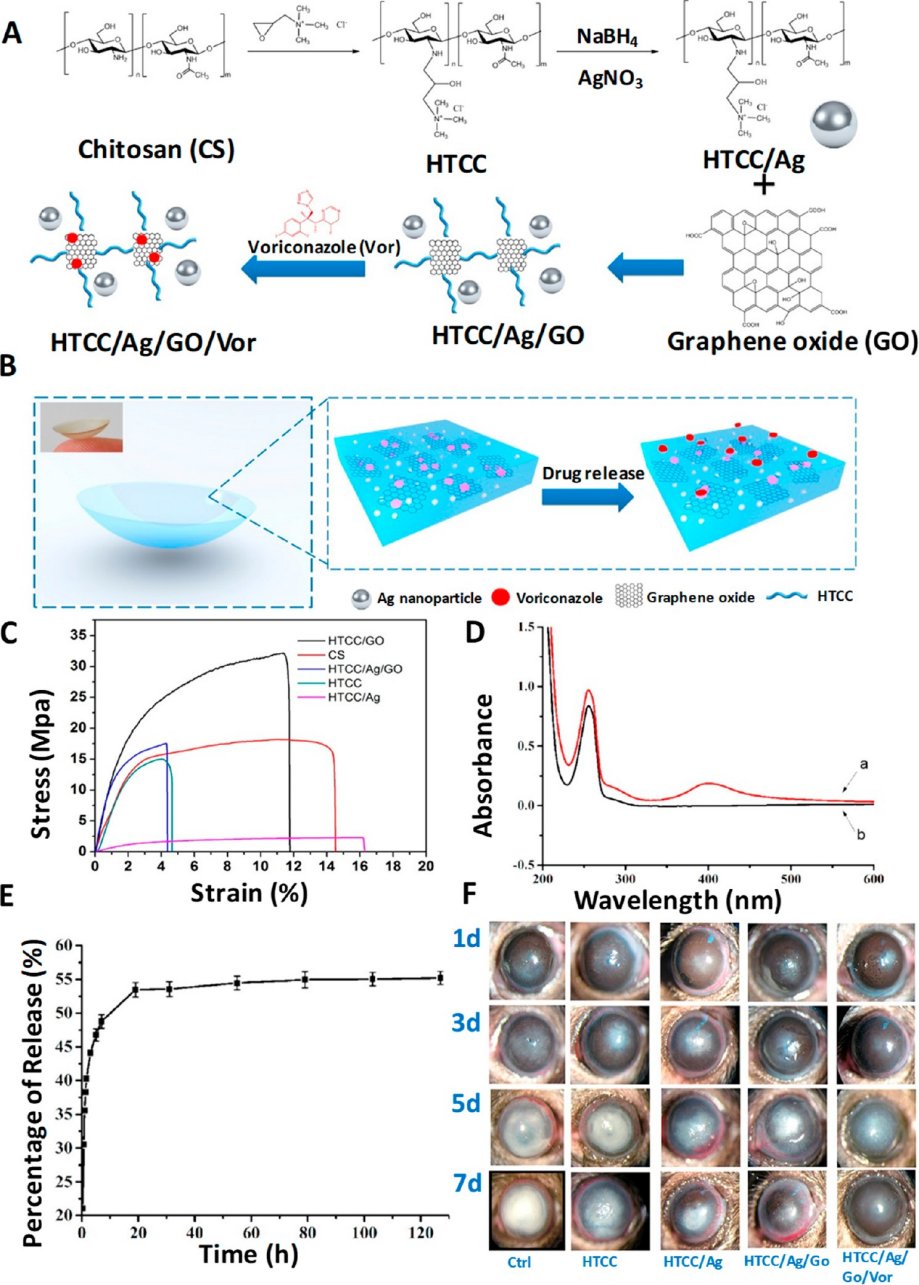
HTCC/Ag/GO membrane embedded contact lens for controlled drug delivery: fabrication, mechanism, and results. (A) Synthesis of HTCC/Ag/GO/Vor. (B) Schematic illustration of drug-loaded contact lenses and controlled drug release. (C) Stress–strain behavior of membranes of CS, HTCC, HTCC/Ag, HTCC/GO, and HTCC/Ag/GO. HTCC and HTCC/Ag were mixed with 5% CS solution in the process of casting of membranes, while HTCC/GO and HTCC/Ag/GO were not. (D) UV–vis spectra of HTCC/Ag/GO/Vor and Voriconazole in PBS: (a) HTCC/Ag/GO/Vor; (b) Voriconazole. (E) Accumulated release curve of Vor in PBS (pH7.4). (F) Photographs of mouse eyes indicating the disease progression at 1, 3, 5, and 7 days. Reprinted with permission from ref (37). Copyright 2016 American Chemical Society.

The adherence of a contact lens’ surface onto the cornea of the eye and its drug loading capacity makes them a suitable device for ocular drug delivery apart from vision correction and diagnostics purpose; particularly, hydrogel-based contact lenses are a suitable platform, offering to carry and release the drug at a sustained rate and assuring superior treatment outcomes with minimized side effects. Chitosan (CS), a naturally occurring polysaccharide, is both biodegradable and biocompatible^223^ and facilitates chemical modifications^183,224^ capable of accommodating drugs. Nevertheless its hardness and rigidity forbid CS from being employed for drug delivery. Therefore, quaternized CS (HTCC) has been an ideal candidate since it has antimicrobial potency due to the positive charge on it. Huang *et al.*(37) demonstrated *in vitro* and *in vivo* studies using HTCC-based hydrogel contact lenses to treat fungal keratitis on mouse. The contact lens was made of the hydrogel, consisting of HTCC, graphene oxide (GO), silver nanoparticles (Ag NPs), and Vor as shown in Figure 17A,B, where HTCC and Ag NPs contribute to the antimicrobial activity of the lens and GO functions as the drug carrier. The GO boosts the mechanical property of the HTCC/Ag/GO hydrogel, enhances the drug loading capacity, and sustains drug release through the π–π stacking interaction between GO and the benzene ring of Vor. The increased mechanical performance of HTCC/Ag/GO is a prerequisite for a theranostic contact lens to treat fungal keratitis.

Both HTCC/GO and HTCC/Ag/GO membranes exhibited superior mechanical performance compared to those of CS, HTCC, and HTCC/Ag (Figure 17C) due to the addition of GO which establishes a strong H-bond with HTCC, or in other words GO supplies carboxylic anion to form electrostatic cross-linking with the cation present on HTCC. UV spectroscopy was used to estimate the quantity of Vor loaded onto HTCC/Ag/GO. In Figure 17D the peak at 255 nm in the UV spectrum confirms the loading of Vor onto the membrane and its amount, and Figure 17E shows the release profile of the drug from the contact lenses to the cornea at pH 7.4 (37 °C). The *in vitro* drug release had been carried out in three phases; the first phase lasted for the first 3 h with an initial burst release of 35% of the drug due to fast diffusion; the second phase lasted for a period of 21 h, releasing 52% of the drug *via* a near-zero-order release; and the third phase after 24 h was a slow one.

Besides voriconazole, natamycin is another FDA approved drug to treat fungal keratitis. Since administering it through intravenous or subconjunctival injections does not guarantee therapeutic concentration, it requires frequent administration in the form of eye drops, which has to last for a week due to its poor penetrability across the cornea.^222,225,226^ Natamycin has water solubility and is highly sensitive to light,^227^ and therefore loading natamycin onto a contact lens is very much hindered and the contact lens with conventional drug loading had a very poor drug release profile of 1 h.^204^ Therefore, core–shell structured poly(d,l-lactide)–dextran nanoparticles (Dex-*b*-PLA NPs)^204^ with hydrophobic PLA as core and hydrophilic dextran as outer shell, obtained by nanoprecipitation, are utilized as the drug carrier vehicle.^228^ Being light-sensitive, natamycin is susceptible to degradation and therefore the contact lens made of polymers such as poly-HEMA and *N*,*N*-dimethylacrylamide is immersed in a solution of natamycin-loaded Dex-*b*-PLA NPs for a week. These contact lenses showed a reduced burst release by 21–54% and an extended release lasting up to 12 h. This polymeric vehicle assisted drug loading by simple soaking method exhibited better loading profile and sustained delivery of the drug.

### Polarity-Based Polymer Vehicle Assistance for Hydrophobic Drugs

Loteprednol etabonate (LPE) is another drug with poor water solubility (0.5 μg/mL)^229−231^ used for treating conjunctivitis, uveitis, and postcataract surgical inflammation, *etc.* Its low solubility in water makes it challenging to load into contact lenses, however, its loading capacity can be improved with the help of lactone (PCL), a hydrophobic biomaterial.^232,233^ A three-layered core–shell nanostructure consisting of a hydrophilic outer shell (PEG), a hydrophobic inner shell (poly-HEMA), and a hydrophobic core (PCL) is prepared by surfactant-free miniemulsion polymerization (SFEP). Then free radical polymerization was employed to prepare HEMA/*N*-vinylpyrrolidone (NVP) based hydrogels with LPE-loaded nanoparticles. The nanoparticles embedded hydrogels showed appreciable transparency even with 15% of nanoparticle loading while the therapeutic contact lenses fabricated out of the nanoparticle-loaded hydrogels sustained release of the drug for an extended period of 12 long days.^234^

Similarly, other drugs such as dexamethasone (DMX)^235,236^ and Prednisolone^237,238^ are the other hydrophobic glucocorticoids used as anti-inflammatory drugs. However, they suffer from poor loading efficiency, which needs a particular method to get embedded into hydrophilic contact lenses. Though Prednisolone has lower glucocorticoid potency than dexamethasone, it exhibited higher corneal permeability.^239,240^ In the case of DMX, ionic interactions^241^ between DMX and chitosan, an *N*-deacetylated of chitin, improved drug loading capacity. Chitosan, known for its biocompatibility and biodegradability,^242−244^ is a cationic polysaccharide polymer whose positive charge can interact with the negatively charged DMX and be incorporated into poly-HEMA contact lenses. The drug-loaded contact lens retained its transparency and exhibited a prolonged DMX release for 22 days.^241^ In order to enhance the loading capability of Prednisolone, poly(lactic-*co*-glycolic acid) (PLGA) nanoparticles^245^ have been used as load vehicle. PLGA is both biodegradable and biocompatible, and it has glycolic acid a hydrophilic entity and lactic acid a hydrophobic entity. The properties of the polymer depend on the ratio between glycolic acid and lactic acid. Oil-in-water (O/W) microemulsion was employed to prepare Prednisolone-loaded PLGA nanoparticles that were incorporated into HEMA/MAA copolymer-based contact lenses. The fabricated contact lenses with Prednisolone were found with a decline in their transparency upon addition of the nanoparticles, while the mechanical properties and surface wettability were retained.^246^ Therefore, cost-effective and sustained drug can be materialized using contact lenses by adopting different strategies.

## Conclusion and Future Prospects

Good health demands close monitoring of many physical parameters on a regular basis since they are indicators of several diseases. Diabetes and glaucoma are two of the deadly diseases that can cause irreversible vision loss if timely attention is not paid.^247^ They both remain dormant and progressively reach a severe state without manifesting symptoms. Glucose concentration and intraocular pressure are the indicators of diabetes and glaucoma, respectively, and monitoring them continuously is inevitable for early diagnosis to avoid adverse effects.^3,47^ These stringent diagnostic measures have not been satisfactorily met by the traditional techniques. The presence of biomarkers in tear fluid extended the role of contact lenses beyond ophthalmic care. The contact lens based sensors are truly non-invasive technology with the ability for continuous monitoring. This new class of sensors has undergone several stages of evolution in their architecture, method of operation, and modes of supplying the readouts. Microfluidic technology eliminated the physical sensing components and offered the sensing platform by manipulating liquids in the picoliter range or a change of color within the engraved microstructure.^119,248^ Looking at the future, the usage of sound waves for measuring IOP can also prove to be an underexplored area with massive developmental potentials, especially due to the low cost of components required for such measurements and the complete non-invasive nature of this method.

In the event of wearable medical applications smart contact lenses turn out to be an excellent convergence of diagnostics and drug delivery. In addition to diagnosis, drug delivery for certain diseases can be also administered using soft contact lenses, which is advantageous over conventional eye drops in terms of extended residence time and ocular bioavailability. Therefore, the focus on soft contact lens based ocular drug delivery is to increase ocular residence time and to minimize anatomical and physiological barriers in order to lower side effects and to boost ocular bioavailability.^210^ To alleviate the poor drug loading and quick release of the drug by conventional soaking method, molecular imprinting methods involving incorporation of polymer nanoparticles have been employed. Polymeric nanoparticles are engaged as drug carriers and incorporated into a hydrogel matrix after being loaded with drug, and in the case of light-sensitive drugs, drug free polymeric nanoparticles incorporation precedes drug loading by soaking, to avoid decomposition of the drug. Depending on the polarity of the drugs, the carrier vehicles and the charge-based interactions are designed and developed to ensure effective drug delivery.^201,249^ With the advantage of simultaneously sensing multiple analytes and a mobile phone assisted self-monitoring system, point-of-care diagnostics has been placed on a higher pedestal. The combination of multiple analytes sensing and a feedback-based automated drug delivery system can revolutionize personalized medicine. With increasing awareness on diabetes and glaucoma, along with constant developments in contact lens based sensors and drug delivery systems, this wearable technology will be adopted as a platform for point-of-care diagnostics and personalized medicine in the near future. Furthermore, the recent evolution in the application of contact lenses to provide shielding against electromagnetic interference and dehydration protection^250^ extends potential promises in healthcare.

## Vocabulary

**Biomarker**: Biomarkers are measurable objective medical signs found in body fluids such as tear fluid, saliva, urine, sweat, blood, or tissues. They are indicative of health, disease state, and the pharmacologic responses to a treatment
**Biosensor**: Biosensor is an analytical device that binds to a biological component to produce a signal proportional to the concentration of the biological analyte. It is used to detect the presence or concentration of a biological analyte
**Diabetes**: Diabetes or diabetes mellitus is a metabolic disorder causing high blood sugar owing to either inadequate production of insulin or incompetent cell response to insulin. Diabetes can cause serious health threats such as damage to eyes, nerves, and kidneys and result in irreversible blindness (diabetic retinopathy)
**Intraocular pressure (IOP)**: It is the pressure inside the eye due to fluid and measures in millimeters of mercury (mmHg). Ocular hypertension is a situation when the IOP exceeds normal eye pressure (10–22 mmHg) arising due to excessive aqueous production or inadequate drainage or medication. Untreated IOP can lead to glaucoma and vision loss
**Glaucoma**: Glaucoma is a bunch of eye conditions caused by very high IOP. Glaucoma is often symptomless and can cause vision loss by causing heavy damage to optic nerves
**Point-of-care diagnostics**: It is a platform to carry out quick detection of analytes close to the patient to provide superior diagnosis, continuous monitoring, and disease management. This can reduce the turnaround time and allow for quick decisions regarding medical management with continuous monitoring of health parameters.
